# bHLH106 Integrates Functions of Multiple Genes through Their G-Box to Confer Salt Tolerance on *Arabidopsis*

**DOI:** 10.1371/journal.pone.0126872

**Published:** 2015-05-15

**Authors:** Aftab Ahmad, Yasuo Niwa, Shingo Goto, Takeshi Ogawa, Masanori Shimizu, Akane Suzuki, Kyoko Kobayashi, Hirokazu Kobayashi

**Affiliations:** Laboratory of Plant Molecular Improvement, Graduate School of Nutritional and Environmental Sciences, University of Shizuoka, 1–52 Yada, Suruga, Shizuoka 422–8526, Japan; University of Western Sydney, AUSTRALIA

## Abstract

An activation-tagging methodology was applied to dedifferentiated calli of *Arabidopsis* to identify new genes involved in salt tolerance. This identified *salt tolerant callus 8* (*stc8*) as a gene encoding the basic helix-loop-helix transcription factor bHLH106. *bHLH106*-knockout (KO) lines were more sensitive to NaCl, KCl, LiCl, ABA, and low temperatures than the wild-type. Back-transformation of the KO line rescued its phenotype, and over-expression (OX) of *bHLH106* in differentiated plants exhibited tolerance to NaCl. Green fluorescent protein (GFP) fused with bHLH106 revealed that it was localized to the nucleus. Prepared bHLH106 protein was subjected to electrophoresis mobility shift assays against E-box sequences (5'-CANNTG-3'). The G-box sequence 5'-CACGTG-3' had the strongest interaction with bHLH106. *bHLH106*-OX lines were transcriptomically analyzed, and resultant up- and down-regulated genes selected on the criterion of presence of a G-box sequence. There were 198 genes positively regulated by bHLH106 and 36 genes negatively regulated; these genes possessed one or more G-box sequences in their promoter regions. Many of these genes are known to be involved in abiotic stress response. It is concluded that bHLH106 locates at a branching point in the abiotic stress response network by interacting directly to the G-box in genes conferring salt tolerance on plants.

## Introduction

Salinity is a major abiotic stress that affects crop production, yield, and growth of plants. Plant response to salinity is well characterized at the cellular, molecular, and physiological levels. Salt stress regulation is a complex process involving numerous changes such as activation of gene expression, transient increase of abscisic acid (ABA) levels, and accumulation of osmoprotectants, antioxidants, and protective proteins [1, 2]. The products of stress induced genes are not only involved in stress tolerance but also in the regulation of gene expression, and in signal transduction of the response [3–5]. Regulatory transcription factor (TF) levels depend upon their own expression patterns, and such autonomous control may be crucial for their transcriptional and post-transcriptional level. The phosphorylation of regulatory proteins is another event that controls gene expression [1]. Furthermore, multiple protein/protein and DNA/protein interactions are known to regulate transcription rates through activation/repression of stress inducible promoters.

Transcriptional regulation is a fundamental process of gene regulation during stress signal responses [6]. Transcriptional regulation of salt responsive genes is a crucial response for plants, and is dependent upon the interaction of TFs with *cis*-regulatory sequences. Although, *cis*-elements of dehydration- and ABA-responsive genes have been the subject of much research, their understanding remains limited [1]. Presently, two kinds of drought responsive elements are well studied: ABA-responsive element (ABRE) and drought responsive elements (DRE) [7]. Plants require a large number of TFs to regulate the response of a given stress, with over 5% of the *Arabidopsis* genome bestowing more than 1,500 TFs [8]. In the control of transcription, TFs bind DNA in the nucleus and interact with basal transcription machinery; stresses may therefore affect either one or a combination of processes. To date, analysis of TFs has revealed that different stress signaling pathways overlap or converge at specific points. Studied TFs include basic leucine zippers [9], homeodomain-leucine zippers [10], Zn-finger proteins [11], AP2/ERF-type TFs [12], Myb-like proteins [8, 13, 14], Myc-like proteins (basic helix loop helix: bHLH) [15, 16], and CDT1 [17].

In recent years numerous efforts have attempted to characterize Myc or bHLH TFs involved in the regulation of different stresses in different species [15, 18–21]. bHLH is a group of functionally diverse TFs, and are well characterized, especially in mammals [22–24]. In animal systems, bHLH TFs have been classified into six main groups (A to F), and are known to play important roles in the control of cell proliferation, development of specific cell lineage in mammalian system, anthocyanin pigmentation, globulin expression, and phytochrome signaling [16, 22–27].

In *Arabidopsis*, bHLH TFs are one of the largest TF families, containing 162 bHLH coding genes classified into 21 subfamilies [16]. The bHLH signature domain consists of approximately 60 amino acids, with two functionally distinct regions. The basic region involved in DNA binding consists of 15 amino acids with higher contents of basic residues, and it is located at the N-terminal end of the domain. The HLH region mainly consists of hydrophobic residues forming two amphipathic alpha helices separated by a loop region of variable length [28]. This region is located at the C-terminal end of the domain and functions in a dimerization form [29, 30]. Structural analysis has demonstrated that HLH regions may function in either homodimer or heterodimer forms, however, it is the basic region of each partner that binds the DNA recognition sequence [31, 32]. The core DNA site recognized by bHLH TFs contains a consensus core element known as an E box (5′-CANNTG-3′), with one of the most common forms being the palindromic G-box (5′-CACGTG-3′). It has been reported that bHLH proteins may even interact with proteins without bHLH domains. Specifically, interactions with MYB, bHLH, and WD40 during the regulation of guard cells and root hair differentiation have been proposed [33].

Several bHLH family genes have been implicated in stress response: *AtICE1* in freezing tolerance [34], *AtMYC2* and *AtAIB* in ABA signaling [15, 20], *AtNIG1* crucial for salt stress signaling [18], *AtbHLH92* in osmotic stress [35], *OsbHLH1* in cold response [21], and *OsRERJ1* in wound and drought responses [19]. Co-expression of *AtbHLH17* and *AtWRKY28* confered resistance to abiotic stress in *Arabidopsis* [36]. Furthermore, overexpression of the most differentially expressed TF gene, *MtbHLH–658*, in a salt-tolerant genotype of *Medicago truncatula* allowed its reference genotype plants to maintain their root growth under salt stress [37]. The function of bHLH TFs would underlie the regulation of expression of target genes involved in salt tolerance. In this investigation, it is characteristic of the selection of salt tolerant mutants by activation tagging among dedifferentiated calli, which lack the differentiated structures such as stomata, trichomes, and root hair as described as known mechanisms of bHLH TFs for salt tolerance [38]. In the mutant, a gene named *salt tolerant callus 8* (*stc8*; *AtbHLH106*) was activated both with and without stress. Here we show that bHLH106 is involved in cold, salt, ABA, and drought stress. Knockout plants were sensitive to different levels of NaCl, and overexpression lines were tolerant to NaCl. Furthermore, bHLH106 could specifically regulate diverse groups of genes related to ABA, ethylene, jasmonic acid, ion transport, and protein phosphorylation and dephosphorylation in different stresses such as cold and salt.

## Materials and Methods

### Transformation and Selection of Mutants

The transgenic *Arabidopsis thaliana* (ecotype Col-0) line 2-1-6, harboring one copy of the pGA-cab-luc-rbcS-gus and pGA-cab-bar-rbcS-hph construct on chromosomes 4 and 5, respectively [39], was used for the generation of activation-tagged mutant lines. Unless otherwise indicated, seeds were surface-sterilized, stratified at 4°C for 1 week, and then sown onto a solidified Murashige-Skoog (MS) medium containing 0.2% Gellan gum (San-Ei Gen F.F.I., Inc., Toyonaka, Japan) [40]. Following 7 days of incubation in growth chambers (20°C, continuous fluorescent light), 15–25 seedlings were transferred into flasks containing liquid MS, and subsequently grown with shaking at 80 rpm for 2 weeks. Cultured roots were then detached from green tissues (stem and leaves) and cut into small pieces (3–6 mm). These were then transferred to CIM (MS supplemented with 0.5 μg/mL 2,4-D and 50 ng/mL kinetin) [41] and incubated in the growth chamber under the previously described conditions for 5 days.

The roots were infected with *Agrobacterium tumefaciens* GV3101 harboring pRi35ADEn4, a binary vector for activation tagging, as described by Niwa et al. [39]. Following 1 week of co-culturing, roots were washed with liquid CIM supplemented with 0.1 mg/mL cefotaxime (Sanofi Aventis, Tokyo, Japan). The roots were then incubated on CIM in the presence of 0.2 mg/mL vancomycin (Merck, Osaka, Japan) and 0.1 mg /mL cefotaxime to inhibit the proliferation of *A*. *tumefaciens*, in addition to 0.1 μg/mL chlorsulfuron (Dr. Ehrenstorfer GmbH, Augsburg, Germany) for transformant selection over a 3-week period. Finally, transformed calli were transferred to CIM supplemented with 0.2 mg/mL vancomycin, 0.1 mg/mL cefotaxime, 0.1 μg/mL chlorsulfuron, and 150 mM NaCl. Mutants were repeatedly selected on the medium. Following an initial selection at 150 mM NaCl, secondary selection was performed at 250 mM NaCl.

### Verification of T-DNA Inserts by PCR

Genomic DNA was isolated from calli that grew on CIM containing 0.1 μg/mL chlorsulfuron using Isoplant II (NipponGene, Toyama, Japan) or as previously described [39, 42]. PCR was used to amplify a ~200-bp fragment of P*35S*-*ALS-SU* ^r^ using the primers 35SminiL-fd [43] and ALS22-rv [39]. The PCR products were subjected to agarose gel electrophoresis using 3% (w/v) Agarose21 (NipponGene, Toyama, Japan).

### Determination of Insertion Location on Chromosomes by TAIL-PCR

Genomic DNA was isolated from mutants and TAIL-PCR [44, 45] performed using AD and T-DNA end primers [39, 45] by TGradient Thermocycler (Biometra, Göttingen, Germany). Following tertiary TAIL-PCR, fragments were purified, and then sequenced directly. The flanking sequences obtained were subjected to a BLAST search using the *Arabidopsis* Information Resource (TAIR, http//:www.arabidopsis.org). Finally, specific primers, 5’-ACAGATATGTACAAACCTCACTAG-3’ (T3K9-RB) and 5’-GGAGGAGGAGAACGGTCAAAGCGG-3’ (T3K9-LB), were designed by MacVector ver. 12 (Cary, NC, USA) and used in combination with T-DNA-specific primers, 5’-CGTTCAAGATGCCTCTACCGACAG-3’ (RB2) and 5’-TGGGATTGTGCGTCATCCCTTACG-3’ (LB2), to amplify specific fragments; these were subsequently sequenced to confirm the insertion sites.

### Real-Time PCR Analysis

Total cellular RNA was extracted using an Isogen II (NipponGene) and treated with RNase-free DNase (Takara, Otsu, Japan). RNA was subjected to cDNA synthesis using a First Strand cDNA Synthesis Kit (Roche, Indianapolis, USA), and real-time PCR conducted using a LightCycler Quick System 330 (Roche). For each reaction, 2 μL of diluted cDNA (equivalent to 200 pg of total RNA) was mixed with 10 μL of SYBR green PCR master mix (Takara) and 10 pmol each of the forward and reverse primers (At2g41130-Fd, 5’-CGACTCCGACCAAACATTATTACC-3’; and At2g41130-Rv, 5’-ATGACCGTCGTTTGAATAGTCTCC-3’) in a final volume of 20 μL. The PCR conditions comprised 45 cycles at 95°C for 5 s and 60°C for 20 s. The amplification was followed by a thermal denaturation step to generate dissociation curves, which verified the amplification specificity. As an internal standard, the *ACTIN2* gene *ACT2* [46] was used for normalization of transcript levels.

### Salt Stress Treatment of Calli

Approx. 62,000 calli were selected on 0.1 μg/mL chlorsulfuran, and maintained by subculturing at 3-week intervals over a period of 3 to 4 months. For the stress treatment of calli, wild-type (2-1-6) and *stc1* calli were cultured on CIM supplemented with 0.1 μg/mL chlorsulfuron and either 150 mM or 200 mM NaCl.

### Construction of Transgenic Vector and Generation of Transgenic Plants

The *bHLH106* coding region at At2g41130 was amplified from genomic DNA using primers 5′- CCCGGGGAAAGCTCCACAAACCCCATTA-3′ (forward, *Sma*I site underlined) and 5′- GGTACCTACTTACAACATTTGCTTACACT-3′ (reverse, *Kpn*I site underlined). The coding region was first cloned into pBluescript II KS+ (Stratagene, La Jolla, CA, USA). Following sequencing, the *bHLH106* coding region was cloned into binary vector pBCH1 [47] containing six copies of the enhancer derived from the CaMV 35S promoter to generate pBCH1-35S-AtbHLH106. The construct was transformed into *A*. *tumefaciens* GV3101 by electroporation. Four- to 5-week-old *Arabidopsis* seedlings were then transformed with *A*. *tumefaciens* harboring pBCH1-35S-AtbHLH106 using the floral dip method [48], and transformants selected using their respective antibiotics. The same construct was used to transform root cultures of the 2-1-6 line, and calli were generated on selective plates.

### PCR Analysis of Transgenic Calli and Plants

PCR analysis using 35S promoter-specific primers [39] was performed on DNA samples derived from T_3_ hygromycin-resistant seedlings and transgenic calli to confirm the presence of the 35S promoter.

### Salt Stress Treatment in Retransformed Plants and Calli

Three-week-old 2-1-6 lines and transgenic calli were transferred to CIM plates supplemented with 150 mM NaCl. After 3 weeks, growing calli were transferred to new plates. Plant seeds were surface-sterilized, and placed in the dark for 7 d. Finally, the seeds were planted on MS medium containing 100 mM or 125 mM NaCl. Specimen photographs were taken 2 to 3 weeks after the transfer to stress medium. *Arabidopsis* were grown on MS medium supplemented with 2% sucrose for 4 days and then transferred to MS medium containing 100 mM or 125 mM NaCl without sucrose.

### bHLH106-GFP Construct and Subcellular Localization

35S promoter was taken from pBCH1 [47] by digestion with *Hin*dIII and *Kpn*I, and cloned in pBluescript II KS+ to make pBS-35S. The *bHLH106* open reading frame (ORF) was amplified with primers, 5’-CCCGGGGAAAGCTCCACAAACCCCATTA-3’ (forward, *Sma*I site underlined) and 5’-GGTACCCACCATGGTGATGTGATCCAGCGCACGAC-3’ (reverse, *Kpn*I and *Nco*I sites underlined) from cDNA prepared from *Arabidopsis* as described below, and cloned at *Sma*I and *Kpn*I sites in pBS-35S to complete pBS-35S-bHLH, followed by sequencing it. A sGFP fragment was prepared from pblue-sGFP(S65T)-nos3’ SK (sSK) [49] and cloned it at *Nco*I and *Kpn*I sites of pBS-35S-bHLH to make pBS-35S-bHLH-sGFP. This plasmid was purified from *E*. *coli* cells using Plasmid Midi Kit (Qiagen, Hilden, Germany) and employed for bombardment of onion epithelial cells by Biolistic PDS-1000/He Particle Delivery System (Bio-Rad, Richmond, CA)

### Whole Genome Expression Analysis

For genome-wide expression analysis of mutants, OX lines, KO lines, and the wild-type, total RNA was extracted from calli using an RNeasy Plant Mini Kit (Qiagen), and then quantified using Agilent 2100 Bioanalyzer (Santa Clara, CA, USA). Two micrograms of total RNA was subjected to cDNA synthesis by One-Cycle cDNA Synthesis Kit (Affymetrix, Santa Clara, CA, USA), followed by purification by GeneChip Sample Cleanup Module (Affymetrix) and labeling with biotin by GeneChip IVT Labeling Kit (Affymetrix). The resultant labeled cRNA was further purified by GeneChip Sample Cleanup Module (Affymetrix), and hybridized with GeneChip Arabidpsis ATH1 Genome Array (Affymetrix) for 16 h according to the manufacturer’s protocol. The hybridized chips were washed and stained using Fluidics Station 450 (Affymetrix) by 49-Format program, and read by Affymetrix GeneChip Scanner 3000 (Affymetrix). Results were analyzed using GeneChip Operating Software (Affymetrix) and with the Partek Genomics Suite 6.6 (St. Louis, MO, USA).

### EMSA

pMAL-c2x (New England BioLabs, Ipswich, MA, USA) was employed to produce MBP-fused bHLH106. The *bHLH106* cDNA was amplified with primers, 5’-GAATTCATGCAACCAGAGACCTCAGATCAG-3’ (forward, *Eco*RI site underlined) and 5’- GGTACCTACTTACAACATTTGCTTACACT-3’ (reverse, *Kpn*I site underlined), and cloned and sequenced in pBluescript II KS+. The bHLH106 cDNA was taken out by digestion with *Eco*RI and *Pst*I, and cloned into pMAL-c2x, followed by transformation in *E*. *coli* strain Rosetta (Merck Millipore, Billerica, MA, USA). The preparation of MBP-fused protein wad done following its manufacturer’s protocol. EMSA was performed with 2 μg of protein in accordance with the methodology and conditions described by Chinnusamy et al. [34].

### Statistical Analysis of Phenotypes

Statistical comparison of degrees of tolerance to salt and other environmental stresses was made on the basis of sequentially rejective Bonferroni (SRB) method in analysis of variance (ANOVA) using js-STAR 2012, http://www.kisnet.or.jp/nappa/software/star/.

## Results

### Identification and Screening of the *stc8* Activation-Tagged Mutant

Numerous efforts have been made to identify salt stress-regulatory components using whole plants [40, 50–53]. This study, however, focused on identification of mutants at the cellular level as the employment of differentiated plants may interfere with analysis, this is due to the variety of complex structures and tissue-specific processes. To investigate salt tolerance at the cellular level, roots of the *Arabidopsis* were infected with *Agrobacterium tumefaciens* harboring the binary vector pRi35ADEn4, this contains four copies of a 339-bp long cauliflower mosaic virus (CaMV) 35S enhancer at the right border of the T-DNA, and a gene for the selectable marker acetolactate synthase as [39]. Transformants were selected on callus-inducing medium (CIM) supplemented with chlorsulfuron, a primary compound of sulfonylurea, and subsequently screened on 150 mM NaCl-containing medium, where calli of the wild-type died. Starting from about 62,000 activation-tagged calli, *18 stc* mutants were identified (see the paper precedent to this article and published together in PLOS ONE, doi: 10.1371/journal.pone.0115502). The mutant line *stc8* which showed the phenotype more significantly tolerant to salt, was subjected to further molecular characterization. T-DNA integration was confirmed by PCR analysis of chlorsulfuron-resistant calli using T-DNA specific primers [39]. The PCR generated a 200-bp fragment, the same size as the product amplified from pRi35ADEn4, thereby confirming the transformation.

### Confirmation of T-DNA in *stc8* through TAIL-PCR

**Fig 1:**
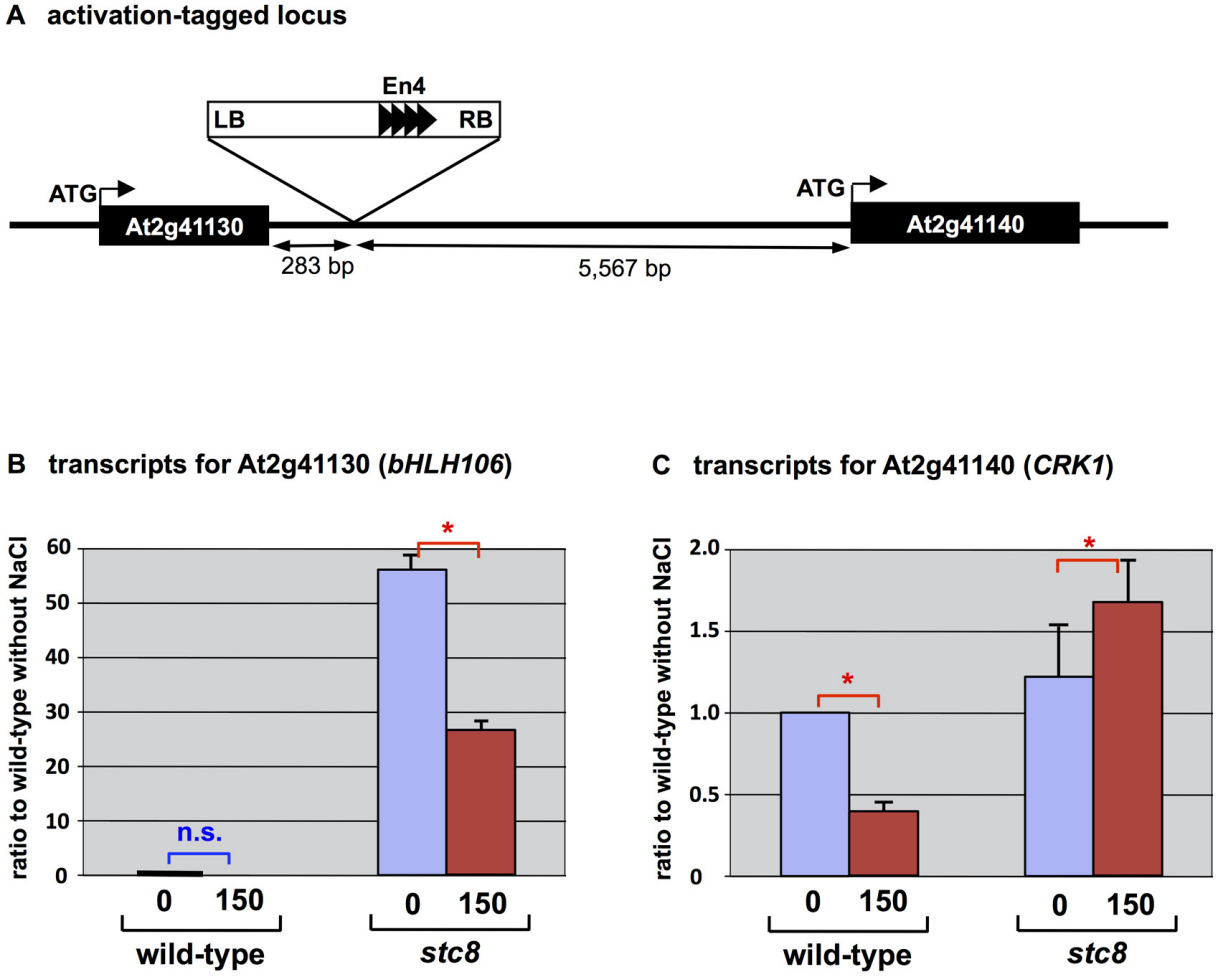
Activation-tagged locus and activated genes in the *stc8* mutant. (A) Integrated location point of T-DNA for activation, in chromosome 2. (B) Expression of At2g41130, encoding *bHLH106* and (C) At2g41140 encoding *CRK1*. Total RNA was extracted from calli grown on CIM supplemented with 150 mM NaCl. Expression was determined by real-time RT-PCR and normalized using *ACTIN2* (*ACT2*) and shown as ratios of transcript levels to those of the wild-type without salt. Error bars represent ±SEM from four experimental replicates. Here are “n.s.” for no significant difference and * for *P* < 0.05 in ANOVA.

To determine the location(s) of T-DNA integration and activated genes, TAIL-PCR was performed for *stc8* mutant using primers from both ends of the T-DNA and arbitrary degenerate (AD) primers [39, 45]. Sequences obtained from TAIL-PCR analyses were subjected to a BLAST search at NCBI, and the location of the T-DNA marked. Specific primers were designed based on the TAIL-PCR sequences, and used in combination with T-DNA-specific primers to confirm the insertion sites within the *Arabidopsis* genome. In the *stc8* mutant, TAIL-PCR confirmed the presence of only one T-DNA, located on chromosome 2. The T-DNA was present between At2g41130 and At2g41140. At2g41130 encoding *AtbHLH106* was located at a distance of 283 base pairs from the border of T-DNA (Fig 1A).

### *bHLH106* is Activated in the *stc8* Mutant

Enhancer sequences within the activation tagging vectors may enhance constitutive or ectopic expression, and the majority of enhanced genes are reported as being located adjacent to the T-DNA [54]. Enhancer sequences may elevate expression at both ends of the T-DNA [55] and are known to enhance gene expression up to 3.6 kbp away, independent of orientation [54]. Transcript levels of genes adjacent to the T-DNA, and 10 kbp flanking regions of the T-DNA, were examined in calli grown on CIM in the absence or presence of 150 mM NaCl. RT-PCR revealed that *bHLH106* transcripts were 27 or 56 times greater with or without 150 mM NaCl, respectively, in the *stc8* mutant in comparison with the wild-type (Fig 1B), and154 times higher in the mutant than the wild-type with salt which dramatically reduced the transcript in the wild-type. Transcripts for another gene locating near the inserted T-DNA, *CDPK-related kinase 1* (*CRK1*), were elevated 1.3 or 1.7 times, with or without NaCl, in the *stc8* mutant compared with the wild-type (Fig 1C), and 2.7 times in the mutant than the wild-type with salt.

### Salt-Sensitive Phenotype of Knockout (KO) Lines and its Rescue by Over-Expression (OX) of bHLH106

**Fig 2:**
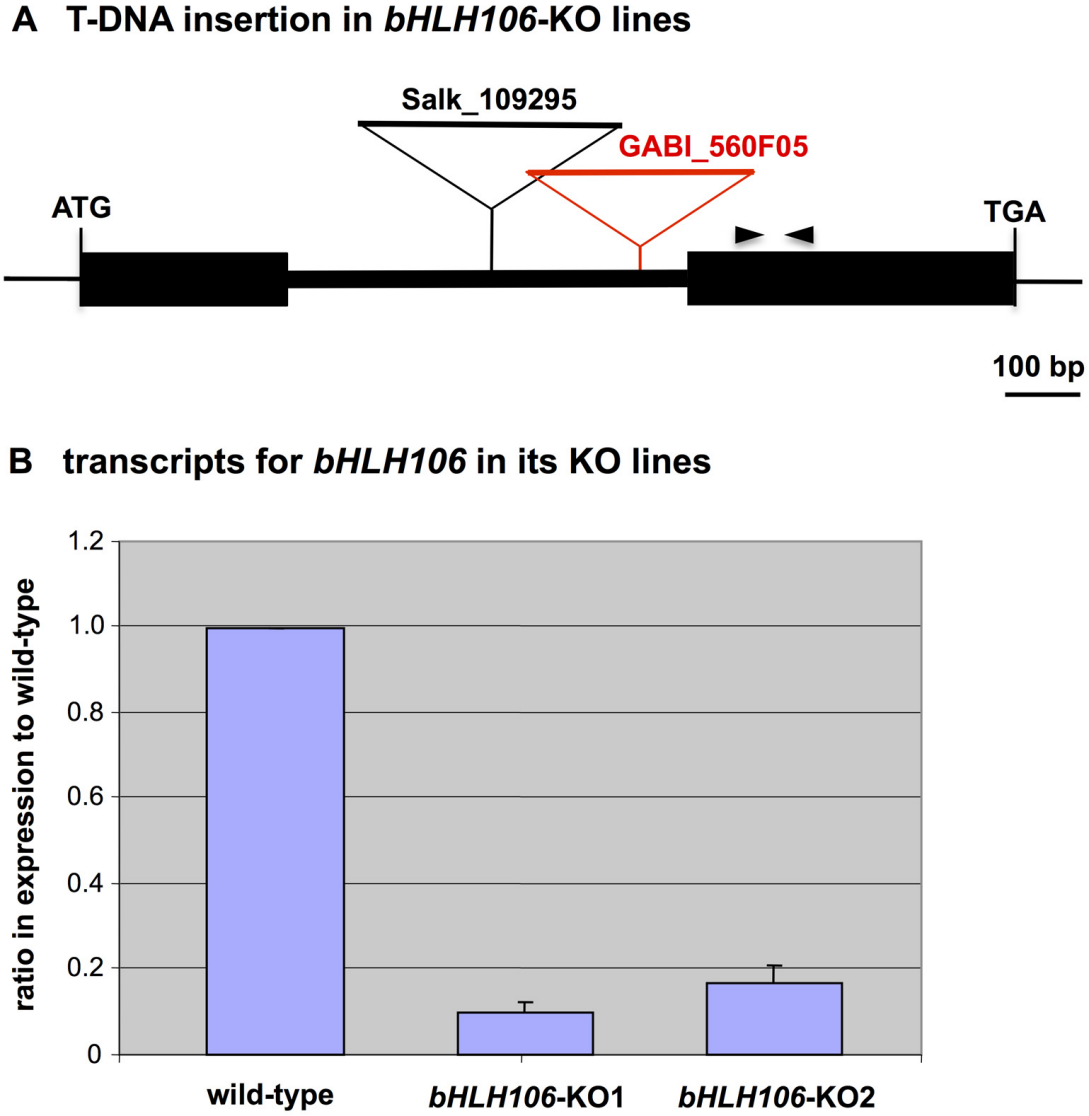
*bHLH106*-knockout (KO) lines. (A) Integrated locations of T-DNA for gene disruption at the *bHLH106* locus in SALK_109295 and GABI_560F05, designated as *bHLH106*-KO1 and *bHLH106*-KO2, respectively. The pair of opposing arrowheads shows the primers employed for RT-PCR. (B) *bHLH106* gene expression in KO plants. Expression was determined by real-time RT-PCR and normalized using *ACTIN2* (*ACT2*). Error bars represent ±SEM from four experimental replicates.

**Fig 3:**
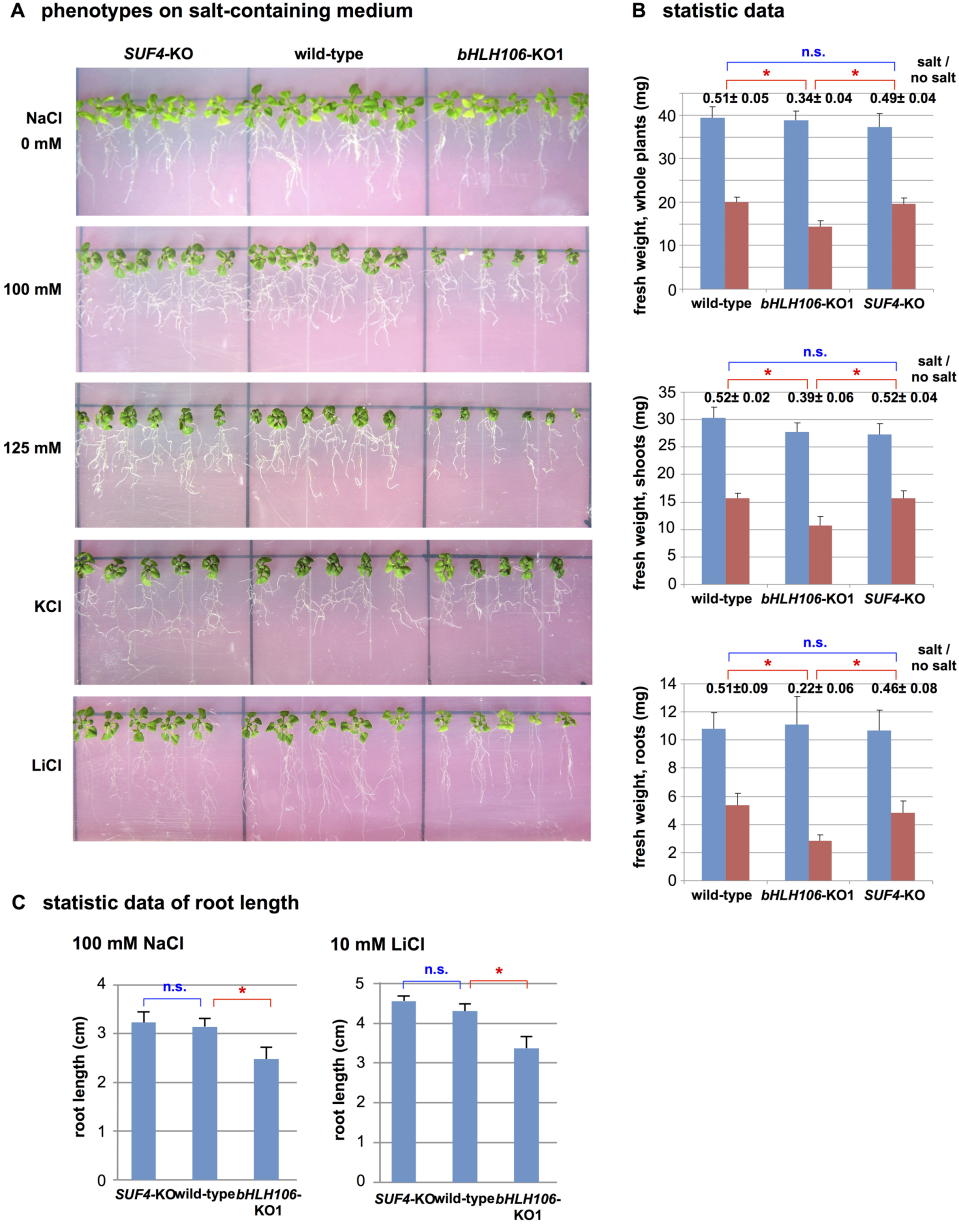
Phenotypes of *bHLH106*-KO line growing on culture medium containing NaCl, KCl, or LiCl. (A) *bHLH106*-KO1 and control plants were germinated and grown on standard MS medium for 4 days, and then transferred to media containing different concentrations of NaCl, 110 mM KCl, or 10 mM LiCl. Plants were then kept vertical in rectangular culture plates for 2 weeks. *SUF4*-KO is a KO line of *SUF4* (At1g30970) for suppressor of FRI4 as an unrelated gene, and was used as a control for KO lines generated by T-DNA at the other loci. (B) Statistic data of plant growth with (red column) or without (blue column) NaCl in the same experiments shown in panel A. Fresh weights of whole plants were measured with or without 125 mM NaCl. Fresh weights of shoots and roots were measured with or without 100 mM NaCl. Error bars represent ±SEM from six experimental replicates. The numbers above columns are ratios of fresh weight of each line with salt to that without salt. Here are “n.s.” for no significant difference and * for *P* < 0.05 in ANOVA of the ratios. (C) Statistic data of root length in the same experiments shown in panel A. Here are “n.s.” for no significant difference and * for *P* < 0.05 in ANOVA.

**Fig 4:**
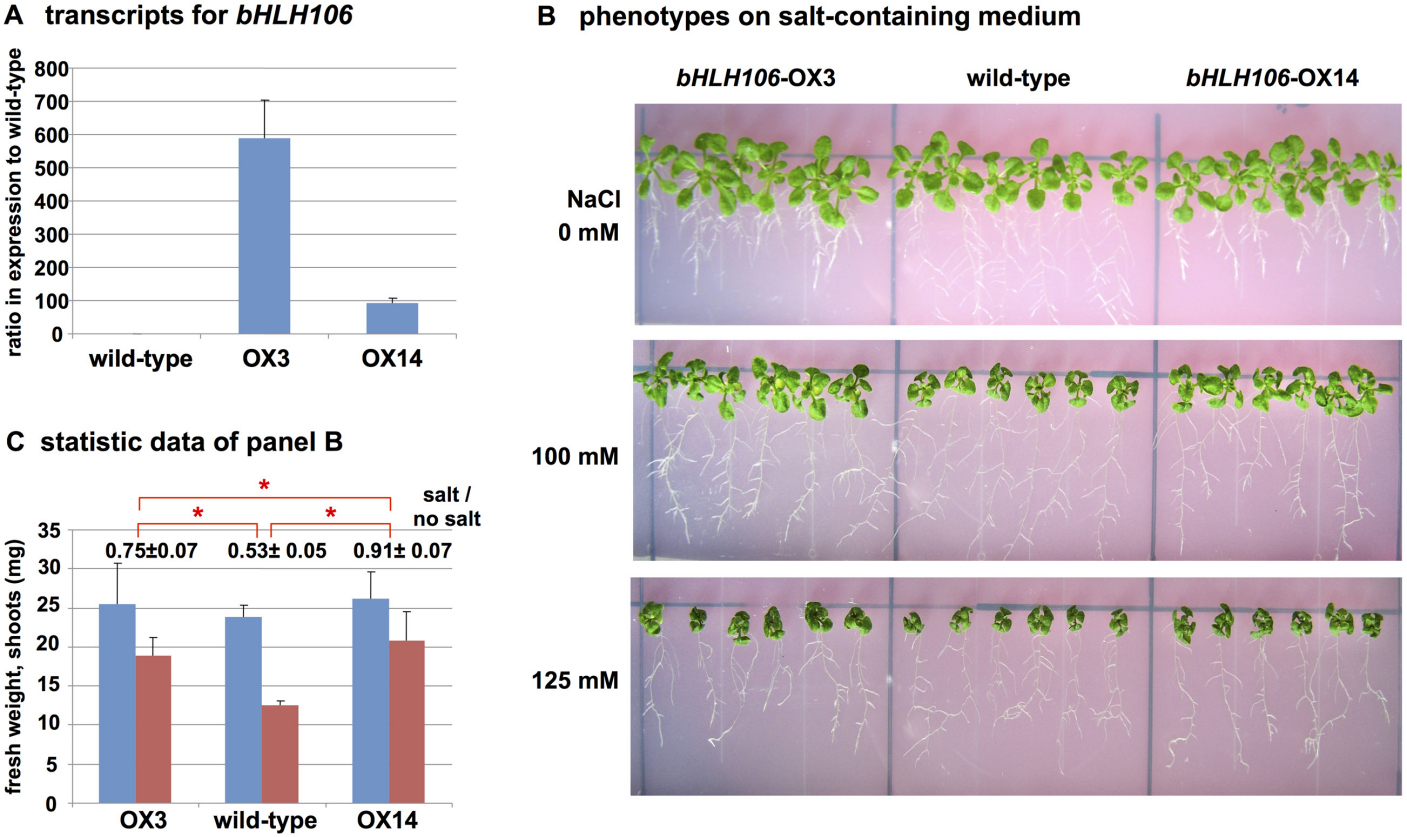
Phenotypes of OX lines of *bHLH106*. (A) Expression of *bHLH106* in OX lines. *bHLH106* was driven by the CaVM 35S promoter and F_2_ homozygous lines used for analysis. Total RNA was extracted from whole plants grown on standard MS. Expression was determined by real-time RT-PCR and normalized using *ACTIN2* (*ACT2*). Error bars represent ±SEM from three experimental replicates. (B) Phenotypes of *bHLH106*-OX grown on culture medium containing different concentrations of NaCl. The culture and treatment of plants with NaCl was as described in the legend for Fig 3. (C) Statistic data of shoot weight of plants growth with (red column) or without (blue column) 100 mM NaCl in the same experiments shown in panel B. Error bars represent ±SEM from six experimental replicates. The numbers above columns are ratios of fresh weight of each line with salt to that without salt. Here is * for *P* < 0.05 in ANOVA.

Two kinds of *bHLH106* KO lines, SALK_109295 and GABI_560F05 (Fig 2A), designated hereafter as *bHLH106*-KO1 and *bHLH106*-KO2, respectively, were analyzed. Transcripts for *bHLH106* were at levels 0.10 and 0.17 fold in *bHLH106*-KO1 and *bHLH106*-KO2 lines compared with the wild-type, respectively (Fig 2B). Homozygous lines of *bHLH106*-KO1 (Fig 3A and 3B) and *bHLH106*-KO2 (S1 Fig) exhibited more sensitive phenotype to salt than that of the wild-type and *SUF4*-KO, which was a knockout line of *SUF4* (*SUPPRESSOR of FRI 4*) encoding a putative Zn-finger-containing TF employed as the control in which an unrelated gene was knocked out. In the *bHLH106*-OX construct, *bHLH106* is driven by the CaMV 35S promoter. The introduction of this construct into the *bHLH106*-KO1 line rescued the phenotype of the KO line in the criterion of leaf extent on salt-containing culture medium (S2 Fig). These results indicated that *bHLH106* caused the *stc8* mutant phenotype. To further confirm that activation of *bHLH106* caused the salt-tolerant phenotype, the wild-type was transformed with the *bHLH106*-OX construct. Transcripts for *bHLH106* were approximately 600 and 100 times higher in *bHLH106*-OX3 and *bHLH106*-OX14 lines than in the wild-type (Fig 4A). The *bHLH106*-OX lines were significantly tolerant to salt with regard to the fresh weights of shoots (Fig 4B and 4C).

### Salt-Inducible and Organ-Specific Expression of *bHLH106*

**Fig 5:**
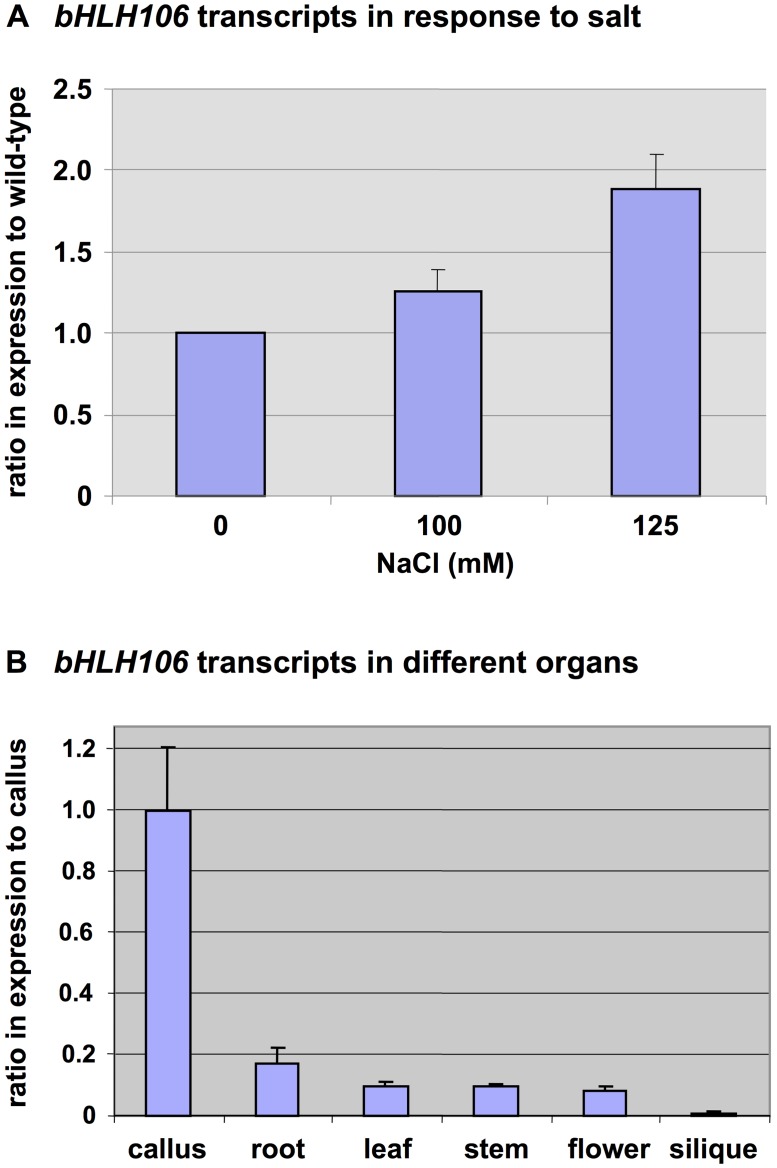
Expression of *bHLH106* with regard to salt-response and organ-specificity. (A) *bHLH106* transcripts in response to salt. Plants were transferred to salt-containing medium 4 days after germination, and kept vertical for 2 weeks. Total RNA was extracted from whole plants and subjected to real-time RT-PCR with normalization using *ACTIN2* (*ACT2*). Error bars represent ±SEM from three experimental replicates. All the *P* values are less than 0.05 between 0 mM NaCl and 100 mM or 125 mM NaCl. (B) *bHLH106* transcripts in different organs. The wild-type plants were grown under standard conditions. RNA was extracted from mature plants. Expression was determined by real-time RT-PCR and normalized using *ACTIN2* (*ACT2*). Error bars represent ±SEM from three experimental replicates.

To further reveal the role of bHLH106 in salt-stress regulation, whether *bHLH106* expression was salt-inducible was examined. Transcripts for *bHLH106* increased 1.3 and 1.9 fold during the 2 weeks after exposure to salt-containing medium (Fig 5A). The order of organ or tissue-specific expression transcription levels of *bHLH106* (from highest to lowest) was calli, roots, leaves, stems, flowers, and siliques (Fig 5B).

### Involvement of *bHLH106* in Response to Environmental Factors

**Fig 6:**
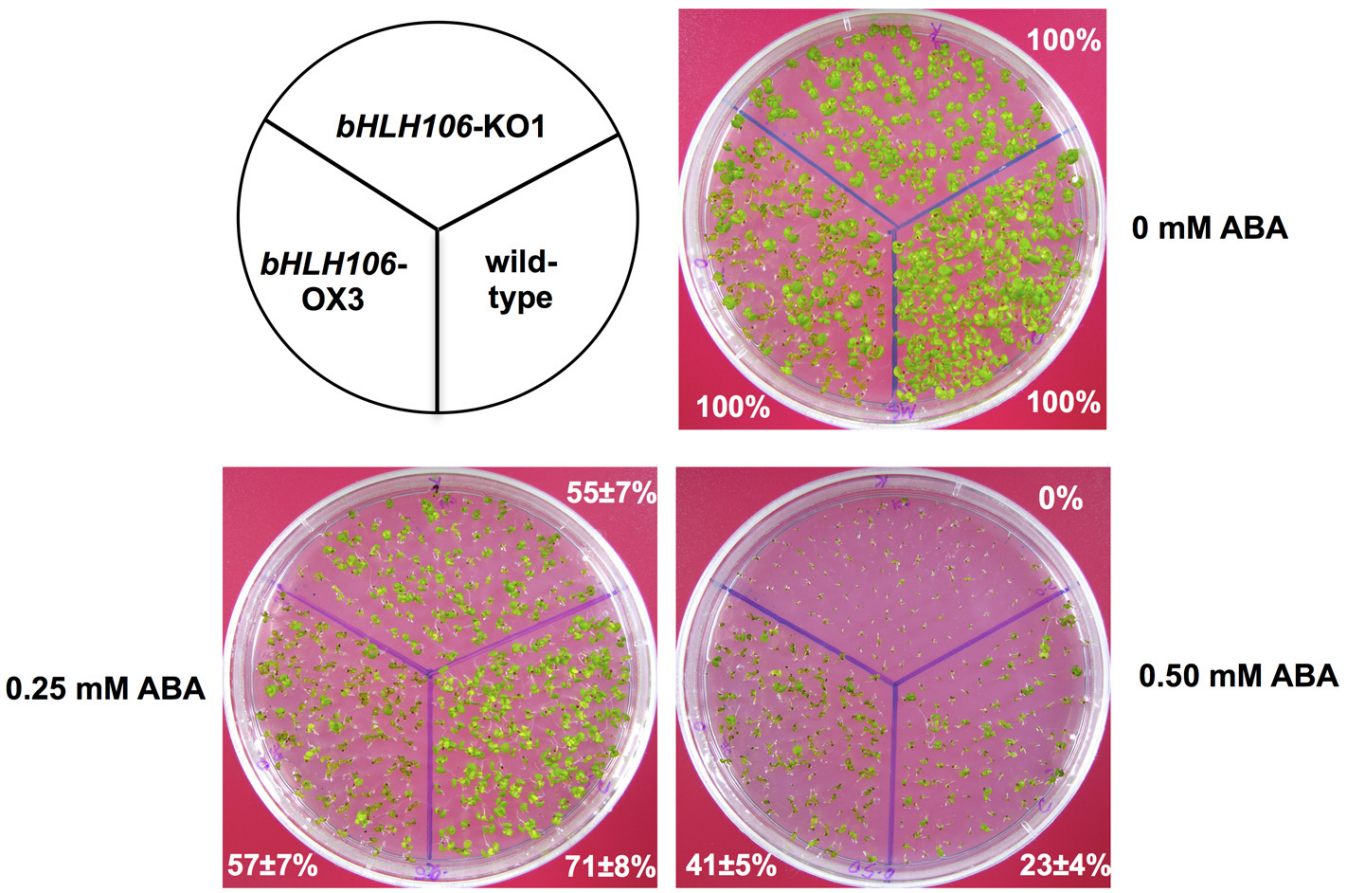
Response of *bHLH106*-KO and *bHLH106*-OX lines to ABA. *bHLH106*-KO1 and *bHLH106*-OX3 lines were grown in medium containing different concentrations of ABA for 2 weeks. The percentages are surviving rates of employed lines in comparison with their survival on the standard MS medium without ABA.

The *bHLH106*-KO lines showed a more sensitive phenotype to 100 mM and 125 mM NaCl, 110 mM KCl, 10 mM LiCl, and treatment at 4°C for 2 weeks (Fig 3A–3C, and S1 Fig). The growth of *bHLH106*-KO1 whole plants was more sensitive to 0.25 mM and 0.5 mM ABA, whereas plants of a *bHLH106*-OX line were less sensitive to 0.50 mM ABA (Fig 6).

### Intracellular Localization of bHLH106 Protein

**Fig 7:**
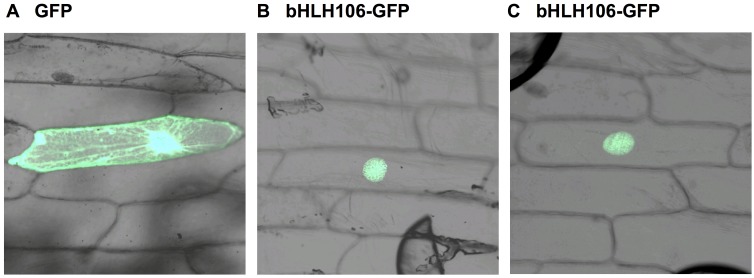
Intracellular localization of bHLH106. The construct of *bHLH106* fused with *GFP* was bombarded into onion epidermal cells. Transient expression of *GFP* was observed. (A) Bombarded with *GFP* alone construct, pTH2 [49, 64]. (B, and C) Bombarded with *bHLH106* fused with *GFP*.

A construct containing bHLH106 fused at its C-terminus to green fluorescent protein (GFP) was transiently bombarded into onion epidermal cells. The fusion protein was located in the nucleus (Fig 7), suggesting that bHLH106 may work as a TF. This intercellular localization is also supported by the typical bHLH structure which may interact with DNA and its function governing the up- or down-regulation of multiple genes in *bHLH106*-OX lines.

### Target *cis*-elements of bHLH106

The hexa-nucleotide E-box sequence, 5′-CANNTG-3′, where the two central nucleotides are variable, is the core DNA motif recognized by most bHLH proteins. One of the most common sequences for the E-box is the palindromic G-box 5′-CACGTG-3′. Within the basic region of proteins, certain conserved amino acids provide identification of the core consensus site, while other residues in the domain dictate specificity for a given type of E-box [56]. Furthermore, flanking nucleotides outside of the hexa-nucleotide core play an important role in binding specificity [22, 57, 58]. There is evidence that a loop residue in proteins plays a role in DNA binding through elements outside of the core recognition sequence [28].

**Fig 8:**
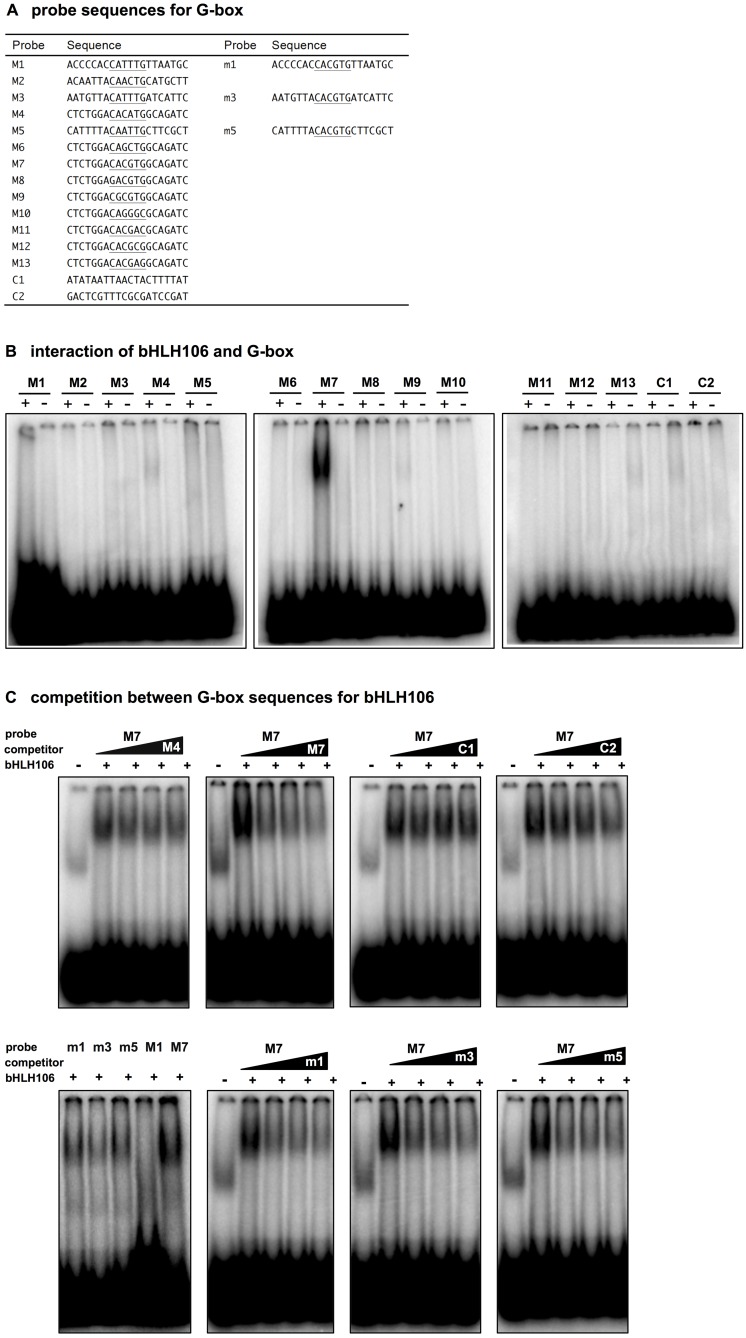
Interaction of bHLH106 with a variety of G-box sequences. Interactions were examined by electrophoresis mobility shift assay (EMSA). (A) Nucleotide sequences of regions containing a possible G-box were employed for EMSA as probes. (B) Thirteen kinds of 20-mer G-box sequences consisting of 5′-^C^/_G_^A^/_G_NN^T^/G/_A_^G^/_C_-3′ (M1 to M13) and arbitrary degenerate probes (C1 and C2). The “+” and “-”denote the presence or absence of bHLH106 protein in EMSA. (C) Competition experiments using the 5′-CACGTG-3′ sequences against the bHLH106 protein.

As AtbHLH106 is a bHLH family TF, it is feasible that its bHLH domain may recognize E-box or G-box core sequences. To determine whether core hexa-nucleotides were recognized by AtbHLH106, a strategy based on ICE1-recognized MYC sequences, all possible combinations of MYC sequences (apMYC), was used (Fig 8A). The CBF3-regulator ICE1 [34], which recognizes five different MYC-sequences present in *CBF3* promoter regions, was used to design all possible MYC combinations of the core six nucleotides recognized by bHLH proteins (Fig 8A).

To produce bHLH106 protein for electrophoresis mobility shift assays (EMSAs), we cloned *bHLH106* cDNA into pMAL as a form fused at its N-terminus to maltose-binding protein, MBP. *Escherichia coli* BL21-Rosetta cells were transformed with the construct, and MBP-bHLH106 purified using maltose binding resin. The apMYC probes and two negative control probes were designed, and annealed using PCR. bHLH106 recognized probe M7, which comprised the 5′-CACGTG-3′ core nucleotides of the G-box, in the EMSA (Fig 8B). No other nucleotide probes interacted with bHLH106, including the controls consisting of arbitrary degenerate sequences. Competition assays using different probes further revealed that bHLH106 recognized the G-box (Fig 8C). To further confirm that bHLH106 recognizes the G-box, 5′-CACGTG-3′ sequence was introduced into probes M1, M3, and M5 to generate m1, m3, and m5 (Fig 8A). All three probes possessing 5′-CACGTG-3′ sequence interacted with bHLH106, and competed with the M7 probe (Fig 8C), demonstrating that bHLH106 specifically interacts with the 5′-CACGTG-3′ sequence of the G-box. The interaction of bHLH106 to m1, m3, and m5 was weaker than seen with M7. Therefore, flanking sequences outside the G-box may affect the strength of interaction. The GC content of flanking sequences likely influences the strength of interaction, as the strongest interaction was observed with M7, which possesses GC-rich flanking regions.

### Genome-Wide Direct Targets of bHLH106

*AtbHLH106* encodes a bHLH protein, which acts as TF. To analyze genome-wide targets of bHLH106, microarray analysis of wild-type and transgenic OX lines of *bHLH106* was performed (see S4 Fig). Out of 22,500 probes present on the ATH1 GeneChip, 551 genes in the OX lines were up-regulated to more than double levels of the wild-type, and 96 genes in the OX lines were down-regulated to levels lower than a half that. These genes were analyzed for the presence of a G-box consensus sequence in their promoter regions to a distance of 3 kbp upstream of each gene. Among the 551 up-regulated genes, 198 genes were direct targets of bHLH106, the position of the G-box(es) present in the promoters were marked (S1 Table). Out of 96 down-regulated genes, 36 had G-box(es) in their promoter region, and were thus directly down-regulated by bHLH106 (S2 Table).

In the computation analysis of the microarray data, 2.44% of all *Arabidopsis* genes were significantly up-regulated by bHLH106, while 0.43% of genes were down-regulated. These results indicate that bHLH106 preferably act as a positive regulator. Following the criterion that they contained G-box sequences in their promoter regions, 35.9% of up-regulated genes and 37.5% of down-regulated genes were directed by bHLH106. Analysis of these up- and down-regulated genes revealed that many were salt, ABA-, jasmonic acid-, and cold-responsive, indicating that bHLH106 plays an important role in multiple abiotic stresses.

Chromosome distribution analyses of the 198 directly up-regulated targets revealed that the maximum number of direct targets of bHLH106 occurred on chromosome 3 (28 targets), followed by 21 on chromosome 1, 18 on chromosome 5, and 16 on chromosome 4. Chromosome 2 had the least targets, with only 7. The 36 down-regulated genes were fairly evenly distributed on chromosomes 1, 3, 4, and 5, with 8, 6, 10, and 7 targets on each respectively. Similarly to the distribution of up-regulated genes, chromosome 2 had the least number of targets (2). Overall, the number of genes positively regulated by bHLH106 was five times greater than that of those down-regulated.

To confirm the direct interaction of bHLH106 with the G-box of up- or down-regulated genes identified from the microarray data of OX lines, we randomly selected 60 promoter sequences (S5 Table). A set of 24-mer probes were designed with nine bases flanking each side of the G-box, and EMSA performed. The EMSA results showed that AtbHLH106 specifically recognized every G-box in the analyzed promoters (S4 Fig). As before, the strength of the interaction signal was influenced by the flanking region sequence. Together, these results indicated that bHLH106 interacts with the G-box in promoters of both positively- and negatively-regulated genes.

### Functional Classification of bHLH106-Directed Genes

**Fig 9:**
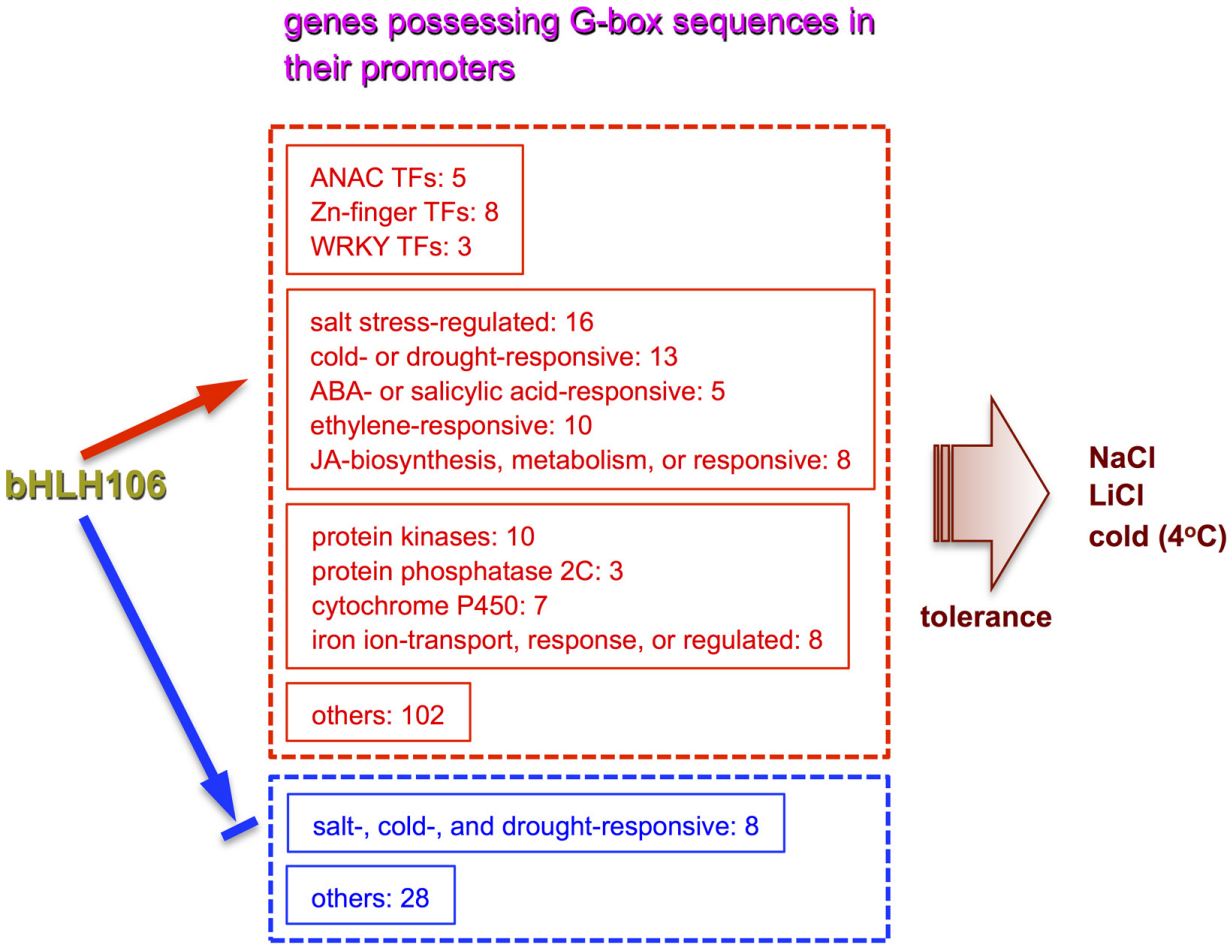
Representation of bHLH106 integrating functions of multiple genes through their G-box to confer salt tolerance on dedifferentiated cells of *Arabidopsis*. bHLH106 directly regulated 198 genes possessing G-box sequences positively, and 36 genes containing G-box ones negatively (S1 and S2 Tables). The up-regulated genes are classified into 12 different groups on the basis of functions (S3 Table). The down-regulated genes are categorized into abiotic stress-responsive ones and the others (S4 Table). The number at each gene group is that of members. The arrow shows positive regulation, and the arrow interrupted with a bar indicates negative regulation. Detailed functions of these genes are described in “S1 Discussion.”

bHLH106 directly regulated 198 genes positively, and 36 genes negatively (S1 and S2 Tables). The up-regulated genes were classified into 12 different groups on the basis of functions: salt stress-regulated genes; cold- or drought-responsive genes; iron ion-transport, response, or regulated genes; protein kinase genes; ANAC TFs; Zn-finger protein genes; ABA- or salicylic acid-responsive genes; ethylene-responsive genes; cytochrome P450 genes; protein phosphatase 2C genes; jasmonic acid-biosynthesis, metabolism, or responsive genes; and WRKY family TF genes (S3 Table, Fig 9). The down-regulated genes included salt-, cold-, and drought-responsive genes (S4 Table, Fig 9).

Many genes related to abiotic stress were positively-regulated by bHLH106, these included the salt stress-regulated genes, salt tolerance Zn-finger 10 (*ZAT10*), salt-inducible Zn-finger 1 (*SZF1*), cupredoxin super family protein, and cation exchanger 3 (*CAX3*); cold- or drought-responsive genes such as dehydration-responsive element-binding (*DREB*) subfamily A5, rare-cold-inducible 2B (*RCI2B*), C-repeat/DRE-binding factor 2 (*CBF2*), a member of the ethylene response factor (*ERF*) / *APETALA 2* (*AP2*) family, DREB1E/dwarf and delayed-flowering (*DDF1*), and ABA-regulated genes such as Ring-DUF1117 and cytochrome *bc*_1_ synthesis (*BCS1*); salt stress related genes such as Ca-dependent protein kinase 28 (*CPK28*) and stress-associated protein (*SAP12*); the jasmonate signal transduction genes jasmonate-ZIM-domain protein 1 and 8 (*JAZ1* and *JAZ8*) and jasmonate-regulated gene 21 (*JRG21*); and the iron transport related genes Fe-regulated transporter 1 (*IRT1*) and ZRT/IRT-like protein 2 (*ZIP2*) (S3 Table). In addition, bHLH106 regulates diverse groups of genes for protein kinases, ANAC TFs, and protein phosphatase 2C (PP2C) (S3 Table). bHLH106 may directly regulate all of these genes under different stress conditions.

Negatively-regulated genes included for small auxin-up RNA (SAUR)-like auxin-responsive proteins, MYB-TFs, and *Arabidopsis thaliana* kinesin 5 (*ATK5*), which is involved in abiotic stress (S4 Table).

## Discussion

### Usefulness of Activation-Tagging of Dedifferentiated Calli

Most previous investigations into the identification of salt stress regulatory components have focused on differentiated whole plants [40, 50–53]. When using the whole plant, the large variety of complex structures and tissue-specific processes may interfere with the elucidation of mechanisms underlying salt tolerance. Salt-overly sensitive 3 (SOS3) and SOS3-like Ca-binding protein 8 (SCABP8) play similar roles with regard to salt tolerance in roots and shoots, respectively [59]. This suggests that it is important to take into account tissue- and organ-specific mechanisms of salt tolerance. In an effort to delineate the mechanisms underlying salt tolerance in cells, this investigation focused on the use of dedifferentiated calli, which possess basal cell functions.

Activation-tagging using enhancer sequences derived from CaMV was applied to select salt tolerant mutants, and further identified causal genes. In the series of this study, we characterized 18 new mutants (named as *salt tolerant callus*, see the paper precedent to this article and published together in PLOS ONE, doi: 10.1371/journal.pone.0115502) showing salt tolerance in dedifferentiated calli. Mutants that were salt-tolerant at cellular levels were selected, and the functions of causal genes in whole plants examined. The advantage of this methodology is that activation-tagging and subsequent screening of dedifferentiated plants allows the skipping of maintenance of established activation-tagged lines.

### Involvement of bHLH106 in Salt Tolerance

The *bHLH106* gene was inducible by salt stress (Fig 5A) and expressed in all organs (Fig 5B). ICE1, a master regulator of cold tolerance, is induced by NaCl, ABA, cold, and dehydration [34]. *bHLH106* expression levels were nearly equivalent among roots, leaves, stems, and flowers, but low in siliques, expression was highest in *in vitro*-cultured calli (Fig 5B). *bHLH106* KO plants were sensitive to NaCl, KCl, LiCl, cold, and ABA (Figs 3 and 6, and S1 Fig), whereas OX lines were relatively tolerant to some of these stresses (Figs 4 and 6), indicating that bHLH106 regulates multiple abiotic stresses. bHLH106 was localized in the nuclei, as revealed by GFP-fusion (Fig 7). These results lead to the conclusion that bHLH106 acts as a positive transcriptional regulator of salt and cold stress.

### The bHLH Gene Family and Functions of its Products

The bHLH TFs are one of the largest TF families in *Arabidopsis*, constituting 9.5% of all TFs, and plays several important roles in plants. Many large families of bHLH TFs have been identified in eukaryotes with sequenced genomes; *Arabidopsis* has the largest relative representation (0.56% of total identified genes; [16]), compared with *Saccharomyces cerevisiae* (0.08%), *Caenorhabditis elegans* (0.20%), *Drosophila* (0.40%), *Takifugu rubripes* (0.40%), human (*Homo sapiens sapiens*; 0.40%), and mouse (*Mus musculus*; 0.50%) [8, 23, 60, 61].

In comparison with animals, few bHLH family TFs have been characterized in plants. The MYC-like TF encoded by the *rd22BP1* gene is induced by dehydration, salt, and ABA [15]. It binds to the MYC-binding site in the promoter region of the *rd22* gene, leading to its expression under drought stress and ABA induction. Similarly, the bHLH gene *OsbHLH1* has been reported to be involved in the cold stress response in rice [21]. The expression pattern of *OsbHLH1* is different from *rd22*, being specifically induced by cold but not by NaCl, PEG, or ABA treatment. In another study, a bHLH encoded by *ICE1* has been characterized as a regulator of the CBF family of TFs, which are known to regulate freezing tolerance [34]. ICE1 binds specifically to MYC-recognition sites in the *CBF3* promoter. Overexpression of *ICE1* provides freezing tolerance in the transgenic plants, indicating that the ICE1 TF is an upstream component of the CBF/DREB1-cold signal transduction pathway, and that ICE1 and CBF/DREB1 belong to the same pathway.

Salt stress adversely affects plant growth and crop production worldwide. Plants invoke numerous mechanisms, involving large sets of genes, to reduce the damage of salt stress. Among these salt-induced genes, TFs play important roles in improving salt tolerance through regulation of genes involved in stress regulation. Although bHLH TFs are predicted to be involved in the regulation of many stresses, their roles in salt stress are not well established. Although their roles in cold tolerance has been the focus of much research [15, 21, 34], further study is required to reveal the mechanism of salt tolerance in detail.

### bHLH106 Binding to the G-Box Regulates Gene Expression

DNA sequences are major determinants of the binding specificity of TFs for their genomic targets. However, eukaryotic cells often simultaneously express TFs with highly similar DNA-binding motifs but distinct *in vivo* targets [62]. bHLH proteins most commonly bind to a DNA motif called the E-box, 5′-CANNTG-3′, first identified as an essential element in the immunoglobulin heavy chain gene regulation [63]. Using yeast TFs Cbf1 and Tye7, it was confirmed that bHLH TF binding sites, *i*.*e*. the E-box, binds differently *in vitro* and *in vivo*, depending on the genomic context [62]. Moreover, computational analyses suggest that nucleotides outside of the E-box binding sites contribute to specificity through influencing the three-dimensional structure of DNA-binding sites [62].

ICE1 binds with five MYC sequences present in the promoter regions of *CBF3* [34]. Based on these MYC sequences and the consensus sequence, we designed 16 probes containing all possible sequence variations (Fig 8A). When EMSA was performed with these probes, based on *ICE1* [34], results revealed that bHLH106 interacted greatest with probe M7, which contained the common palindromic G-box sequence, 5′-CACGTG-3′ (Fig 8). Furthermore, when the G-box sequence was introduced into the outside sequences of non-interacting probes M1, M3, and M5, to generate m1, m3, and m5, respectively, bHLH106 interacted with them, though at lower levels compared with the M7 probe (Fig 8C). Therefore, flanking sequences of the G-box contribute to the interaction with bHLH proteins. The most acceptable explanation regarding the contribution of the flanking sequences is that in probe M7 the next bases to 5′-CACGTG-3′ at the 3′ position are 5′-GC-3′, furthermore the overall GC content of probe M7 is higher than in probes m1, m3, and m5. Conversely, the presence of A or T repeats around the G-box or overall in the probes significantly reduced interaction with the bHLH protein. These explanations were further confirmed when EMSA was performed using 24-bp gene-specific probes of promoters containing the G-box (S3 Fig). The interaction in probes 1, 7, 11, and 18 was low, and could be explained by the absence of G or C next to the G-box, or by the presence of A or T repeats in the probes. Contrarily, probes 2, 3, 4, and 5 exhibited strong interactions with bHLH105, suggesting the importance of the contribution of G or C next to the G-box to the interaction. It is concluded that bHLH106 specifically interacts with 5′-CACGTG-3′ in promoters, and that this specificity is influenced by flanking sequences.

### Signals Diverge at bHLH106 in the Abiotic Response Network

Genes satisfying the criteria of being up-regulated by bHLH106 and having bHLH106-binding G-box sequences present in their promoter regions encode TFs (ZAT10, SZF1, DREB, CBF2, and ANACs), abiotic stress-regulated proteins (CAX3, RCI2B, ERF/AP2, DDF1, Ring-DUF1117, BCS1, and SAP12), protein kinases (CPK28, diverse groups), jasmonate (JA)-related proteins (JAZ1, JAZ8, and JRG21), proteins involved in transport (IRT1 and ZIP2), and protein phosphatase (PP2C) (see “S1 Discussion” for detailed functions of these genes). It is thought that bHLH106 may directly regulate all of these genes under different stress conditions. Therefore, it is concluded that bHLH106 is a key TF, which locates at a branching point for abiotic stress-related signal transduction pathways (Fig 9).

## Supporting Information

S1 Discussion Discussion of bHLH106-Targeting Genes. (DOCX)

S1 Fig Phenotypes of *bHLH106*-KO2 line growing on culture medium containing KCl or under cold stress. (A) *bHLH106*-KO2 and wild-type plants were germinated and grown on standard MS medium for 5 days, and then transferred to medium with or without 110 mM KCl. Plants were incubated at 4°C for 2 weeks after transferring to standard MS in rectangular culture plates (4°C), and returned to the culture condition at 20°C for 1 week (4°C > 20°C). (B) Statistic data of root length in the same experiments shown in panel A. Error bars represent ±SEM from six experimental replicates. Here are “n.s.” for no significant difference and * for *P* < 0.05 in ANOVA.
(PDF)

S2 Fig Phenotypes of rescue lines of *bHLH106*-KO1 using the *bHLH106*-OX construct. (A) Expression of *bHLH106* in leaves of the rescue (RES) lines. *bHLH106*-KO1 lines was transformed with a construct for *bHLH106* overexpression (OX) driven by the CaVM 35S promoter. Homozygous lines F_2_ were for analysis. Expression was determined by real-time RT-PCR and normalized using *ACTIN2* (*ACT2*). Error bars represent ±SEM from three experimental replicates. All the *P*-values are less than 0.01 between the wild-type and the rescue lines. (B) Phenotypes of *bHLH106*-RES lines grown on culture medium containing 125 mM NaCl. The culture and treatment of plants with NaCl was performed as described in the legend for Fig 3. (C) Statistic data of leaf extent in the same experiments shown in panel B. Error bars represent ±SEM from six experimental replicates. Here is * for *P* < 0.05 in ANOVA.
(PDF)

S3 Fig Interaction of bHLH106 with 60 kinds of promoter sequences, including the G-box. The locus number of these genes are given in S5 Table.
(PDF)

S4 Fig ANOVA data of transriptomic analysis. (A) Hierarchical clustering. The analyses were done for gene expression in the lines *bHLH106*-OX3, *bHLH106*-OX14, and wild-type in their triplicate or quadruplicate by Partek Genomics Suit 6.6. (B) Volcano plot. The analysis was performed as described for Panel A.
(PDF)

S1 Table Genes satisfying both criteria of presence of G-box in promoters and up-regulation in OX Lines. (DOCX)

S2 Table Genes Satisfying both criteria of presence of G-box in promoters and down-regulation in OX Lines. (DOCX)

S3 Table Functional classification of genes satisfying both criteria of presence of G-box in promoters and up-regulation in OX Lines. (DOCX)

S4 Table Salt-, cold-, or drought-responsive genes which also satisfied both criteria of presence of G-box in promoters and down-regulation in OX Lines. (DOCX)

S5 Table Probes for genes possessing G-box in their promoters. (DOCX)
