# Supplementary material for: bHLH106 Integrates Functions of Multiple Genes through Their G-Box to Confer Salt Tolerance on Arabidopsis
Source: PLoS One. 2015 May 15;10(5):e0126872. doi: 10.1371/journal.pone.0126872 (PMC4433118; doi:10.1371/journal.pone.0126872)
Supplement: S1 Discussion — (DOCX) [file pone.0126872.s001.docx]

**S1 Discussion. Discussion of bHLH106-Targeting Genes.**

**Salt Stress Regulated Genes**

bHLH106 directly regulates 16 salt regulatory genes, these have either direct or indirect roles in salt stress regulation (S3 Table). Among the salt stress regulated genes, expression of *AtHSFA4A* was more than two-fold up-regulated in the *bHLH106* over-expression (OX) lines and contains two G-boxes, located at position 75 and 497 in the promoter region. *HSF2A* was also up-regulated in wild-type plants upon H_2_O_2_ stress [1]. *CAX3* is a cation exchanger differentially regulated by Na^+^ stress [2], and its expression was approximately three-fold upregulated in the *bHLH106* OX lines, it contains three G-boxes in its promoter region, at positions 1057, 1654, and 2302. Additionally, the *CAX3* promoter also had five different additional G-box derivative Myc sequences, further supporting that *CAX3* is regulated by bHLH transcription factors. A member of the DREB subfamily A-5 of the ERF/AP2 (*AtDS1*) transcription factor family was enhanced two-fold, and had two core consensus sequences present at positions 2825 and 2899. A MPK3 and MPK6 interacting zinc finger protein, *ZAT10*, was also positively regulated by *bHLH106*, and contains one core G-box at position 2760. The gene for salt inducible zinc finger protein *SZF1* contains a core G-box at position 2496 of its promoter, and was also positively regulated in the *bHLH106*-OX line. Additional genes include, a cupredoxin family protein, an affinity nitrate transporter, a zinc finger protein 1, *Arabidopsis thaliana* protein kinase 19 (*AtPK19*), a NUDIX hydrolase homolog 7, a DA1-related protein 3 (*DAR3*), and an F-box protein induced by various abiotic stresses. Expression of all of these genes was enhanced in over-expression lines and their promoters all contained G-boxes. Taken together, these results indicate that *bHLH106* positively regulates genes that are important salt stress regulatory components in *Arabidopsis* and has a vital role in the salt stress regulatory network.

**Cold and Drought Responsive Genes**

In the group of cold and drought responsive genes regulated by *bHLH106* (S3 Table), various important genes were up-regulated in the over-expression lines, these included: rare cold inducible 2B (*RCI2B*), C-repeat Binding Factor 2 (*CBF2*), an Ethylene responsive (*ERF*/*AP2 B4* or *RAP2.6L*) transcription factor, a member of the DREB family transcription factor (*DREB A-1* or *DDF1*), and a cold inducible zinc finger protein (*C2H2*). *RCI2B* and *DDF1* both have two G-boxes in their promoter sequence, and their expression level was enhanced more than two-fold in the GeneChip analysis. Expression of *RCI2A* and *RCI2B* was induced by low temperature, salt, drought, and ABA treatments, and both proteins play important roles in various abiotic stresses [3]. DDF1 is also an important regulator of cold and drought responses and enhanced expression of *DDF1* results in improved tolerance to salt, drought, cold, and heat [4].

*CBF2*, *ERF*/*AP2* *B4* (*RAP2.6L*), and *C2H2* (*ZAT6*), which each have one G-box motif in their promoter for *bHLH106*, had their transcript levels activated more than two-fold in the over-expression lines (S3 Table). C-repeat-binding factor (CBF) / dehydration-responsive element-binding factor (DREB1) proteins constitute a small family of *Arabidopsis* transcriptional activators (CBF1/DREB1B, CBF2/DREB1C, and CBF3/DREB1A) that play a prominent role in cold acclimation. Over-expression of *CBF1-3* genes enhances plant frost tolerance, and *CBF2* overexpression suppresses leaf senescence induced by the stress hormone ethylene [5]. Together, these results demonstrate that bHLH106 improves cold tolerance by regulating *CBF2*, *RCI2B*, *DDF1*, and *ZAT6*.

**Iron Ion Transport / Responsive Group**

Iron is an essential element required for cellular functions including photosynthesis and respiration in plants. Iron deficiency poses an agricultural challenge because a lack of iron availability often limits plant growth. Different Iron transporters identified in tomato (*Solanum lycopersicum*) and *Arabidopsis* include FER, FIT1, and POPEYE [6-8]. There were eight differently annotated genes (S3 Table) responsive to iron transport in GeneChip data of the *bHLH106* OX line. Of particular interest are two iron transporters, IRT1 and AtZIP2. *IRT1* expression was approximately four-fold enhanced, and it contains one G-box in its promoter at position 2301. IRT1 is also involved in the transport of Mn, Zn, and Co [9]; IRT1 also complements the Zn-uptake deficient yeast mutant strain (zrt1zrt2). In addition, expression of *IRT1* is also enhanced under iron deficient conditions [9]. Expression of *AtZIP2* was also enhanced two-fold, and it contains one G-box motif at position 298. Members of the ZIP gene family are involved in transport of Cd, Zn, Mn, and Fe. IRT1 and IRT2 are also members of the ZIP gene family [10]. These results demonstrate that bHLH106 regulates *IRT1* and *AtZIP2*, which are known to regulate iron transport in *Arabidopsis*.

**Protein Kinases**

Calcium dependent protein kinases (CDPK) and mitogen activated kinases (MAPK) play important roles in defense, biotic, and abiotic stresses in *Arabidopsis*. *bHLH106* activated nine different members of the protein kinase family, including CDPK, serine threonine kinase, and MAPK (S3 Table). *AtMPK3* and *AtMKK9* were both expressed two-fold in the *bHLH106* OX line, and contain G-box motifs in their promoters. Additionally, ATMPK3 and AtMKK9 are also involved in the salt stress signaling pathway [11, 12]. This is consistent with our results that *bHLH106* activated most of the abiotic stress signaling genes in the GeneChip analysis without stress to the over-expression lines. Moreover, another member of the MAPK family, *AtMPK11*, was activated over three-fold in the overexpression lines, and contained one G-box in its promoter. Pathogen attack or elicitation with pathogen-associated molecular patterns (PAMPs) and stresses activate expression of *AtMPK11* [13]. Constitutive expression of the stress-response transcriptional coactivator multiprotein bridging factor 1c (*MBF1c*) in *Arabidopsis* enhances tolerance to heat, and osmotic stresses that lead to activation of *AtMPK11* [14]. Expression of the protein kinase *AtPK19* increases when plants are subjected to cold and high salinity [15]; this gene was also regulated by *bHLH106* and has two G-boxes present in its promoter region, at positions 1779 and 2916. These results further demonstrate that bHLH106 regulates members of the protein kinase family important for salt and cold stress.

**NAC (ANAC) Transcription Factors**

NAC transcription factors are key regulators of stress perception and developmental programs, and most share an N-terminal NAC domain. The genomes of *Arabidopsis thaliana*, tobacco (*Nicotiana tabacum*), and rice (*Oryza sativa*) all contain over 100 genes encoding NAC transcription factors, making it one of the largest plant gene families. Five members of the NAC family, *ANAC055*, *ANAC047*, *ANAC092*, *ANAC001*, and *ANAC087* were upregulated more than two-fold in *bHLH106* transgenic lines. They all contain one G-box binding motif in their promoters, except for *ANAC055* that has two, at positions 2502 and 2888 (S3 Table). ANAC019 and ANAC055 have a dual role in regulating jasmonic acid (JA) and ABA responses [16]. Both ANAC019 and ANAC055 act downstream of AtMYC2 to regulate JA signaled defense response. AtMYC2 is also a bHLH protein, and acts as an activator in ABA signaling and drought response, and recognizes G-box motifs in promoters [17]. These results suggest that bHLH106 may act in combination with AtMYC2 to regulate ABA and JA-signaling response through ANAC055. ANAC092, another member of the NAC transcription factors regulated by bHLH106 is also salt inducible and is a major promoter of plant senescence [18]. In addition, the majority of genes upregulated by ANAC092 induction are also salt responsive [18].

**Zn-Finger Proteins (ZFPs)**

Zinc finger proteins are a super family of proteins involved in numerous plant growth and development activities. They are known to regulate resistance mechanisms for various biotic and abiotic stresses. Among the 176 ZFPs in *Arabidopsis*, eight members were activated in the over-expression lines (S3 Table). All eight members had one G-box motif that was recognized by bHLH106 in their promoter. Out of these, five members (At1g66500, At3g28210, At4g14365, At5g59550, and At5g67450) had at least three-fold enhanced expression in the *bHLH106*-OX lines (S3 Table). Moreover, the level of *SAP12* (At3g28210) transcript is strongly induced under cold and salt stress in a time-dependent manner similar to that of *SAP10* [19]. In addition, pre-mRNA cleavage complex II (At1g66500) is also stress responsive [20]. Another RING E3 ligase, AtRDUF2 (At5g59550) plays a combinatorial role with AtRDUF1 (At3g46620) and is induced by ABA and drought stress [21]. At3g46620 was also induced in the *bHLH106*-OX lines. The A20/AN1 zinc finger family protein is also responsive to cold, light, and drought [22, 23]. These results provide further evidence that bHLH regulates zinc finger proteins involved in salt stress response and activates a diverse group of genes.

**Ethylene Response Factors**

Ethylene-responsive element binding factors (ERFs) are members of a novel family of transcription factors specific to plants. A highly conserved DNA binding domain known as the ERF domain is the unique feature of this protein family. bHLH106 directly regulates six members of this family (S3 Table), and all six members (RRTF1, ATERF13, ATERF1, ATERF6, ATERF2, and ATERF11) are involved either directly or indirectly in abiotic stress. Redox responsive transcription factor 1 (*RRTF1*) and *AtERF1* each have four bHLH106 binding motifs in their promoter sequences, their expression increased approximately five-fold or three-fold, respectively, in the *bHLH106* transgenic lines (S3 Table). The other four members *AtERF13*, *AtERF6*, *AtERF2*, and *AtERF11* have one G-box in their promoters, at positions 1985, 105, 2829, and 2658 respectively; each member was up-regulated more than two-fold in the over-expression lines (S3 Table). *RRTF1* was upregulated without stress in the *atr7* mutant, which itself is tolerant to oxidative stress induced by paraquat (PQ) or the catalase inhibitor aminotriazole (AT). *RRTF1* was induced in *atr7 loh2* and wild-type backgrounds upon AT and PQ stress, indicating that RRTF1 acts as marker of reactive oxygen species (ROS) [24]. This result is similar to our results, where *RRTF1* expression was enhanced five times without stress in the *bHLH106* OX lines.

ABA is a plant hormone that controls the protective response to various abiotic stresses. The promoters of all ABA regulated genes share a consensus core sequence, 5’-ACGTGGC-3’, and the importance of this core sequence, generally known as an ABA response element (ABRE), has been demonstrated by various experiments. Its related transcription factors, known as ABFs/AREBs, have been identified. Although necessary, ABRE alone is not sufficient for full ABA-regulation of gene expression, with another *cis*-element, known as a "coupling element (CE)" required. AtERF13 was one of the first CE element binding factors (CEBFs) identified and confers ABA hypersensitivity in *Arabidopsis* [25]. These results support our results that bHLH106 regulates important genes involved in the ABA response.

*AtERF1* was up-regulated three-fold in transgenic *bHLH106* OX lines and has four G-box motifs in its promoter (S3 Table), suggesting that *AtERF1* is also controlled by bHLH106. Moreover, AtERF1 is reported to be responsive to both three and twenty-seven hour treatments of cold stress [26]. Three ethylene responsive genes *AtERF6*, *AtERF2*, and *AtERF11* were also responsive to different abiotic stresses.

**Cytochrome P450 Genes**

*Arabidopsis* contains 272 different cytochrome P450 genes, and their biological functions include the synthesis of structural macromolecules such as lignin, cutin, or suberin; the synthesis or catabolism of hormone or signaling molecules; the synthesis of pigments and defense compounds; and the metabolism of xenobiotics. There were seven cytochrome P450 genes (*CYP707A3*, *CYP94C1*, *CYP81D1*, *CYP94B3*, *CYP71A19*, *CYP71A20*, and *CYP71B23*) up-regulated in the *bHLH106*-OX lines. These cytochrome P450 genes had G-box recognition motifs in their promoters and had expression enhanced more than two-fold in the GeneChip data (S3 Table). These Cytochrome P450 genes are involved in many different stresses.

Expression of *CYP707A3* is highly induced in response to both dehydration and subsequent rehydration [27]. *cyp707a3* mutant plants are drought tolerant and have increased ABA inducible gene expression. On the other hand, the constitutive expression of *CYP707A3* relieved growth retardation by ABA, increased transpiration, and reduced endogenous ABA in both turgid and dehydrated plants. Therefore, CYP707A3 plays an important role in maintaining the threshold level of ABA during dehydration and rehydration [27]. These result support our data that *bHLH106* regulates ABA induced expression of *CYP707A3*.

Jasmonoyl-isoleucine (JA-Ile) is a specific ligand that binds to the COI1-JAZ co-receptor, thus relieving repression of JA responses. *CYP94C1* and *CYP94B3* are both wound inducible and involved in JA-Ile oxidation, which is a major catabolic route for JA-Ile turn-over [28]. Additionally, the expression of CYP94C1 is enhanced by stress, treatment with the hormone methyl JA, and wounding [29]. Both *CYP94C1* and *CYP94B3* are important for JA response, and their expression was enhanced three-fold or more in the *bHLH106*-OX lines; both genes contain one G-box motif in their promoters. These results suggest the importance of bHLH106 in the regulation JA responsive genes. *CYP81D8* was up-regulated two-fold in the *bHLH106* OX lines, it has one G-box motif and is also up-regulated in response to NaCl stress [26]. Together, these results indicate that bHLH106 regulates important cytochrome P450 genes involved in ABA, salt, and JA stress.

**Protein Phosphate 2C (PP2C) Genes**

Members of the PP2C group, from various organisms, have been implicated in acting as negative modulators of protein kinase pathways activated by diverse environmental stresses or developmental signaling cascades. In plants, several PP2Cs have been described as negative regulators within the ABA mediated signaling network. *bHLH106* may regulate one, two or all three members of the *Arabidopsis* PP2C family (At2g05050, At3g27140, and At4g08260) because a single probe in ATH1 represents all three genes (S3 Table). Expression of this probe was upregulated more than two-fold. Interestingly all three genes had their G-box motif at exactly the same position (2853) in the promoter, despite being on different chromosomes (S3 Table). PP2C are emerging players in the regulation of abiotic stress responses, and especially of ABA signaling in plants [30]. In eukaryotes, one of the roles of PP2Cs is to reverse stress induced protein kinase cascades. PP2Cs cooperate with other types of phosphatases (PTPs, DSPTPs, and PP2As) in the dephosphorylation of core components of signal transduction cascades such as RLKs, MAPKK kinases, MAPKKs, and MAPKs. The above three PP2C members belong to group B of the protein phosphatases, and are characterized by their homology to MP2C, an alfalfa PP2C that regulates MAPK signaling. As the above PP2Cs were up-regulated in the transgenic *bHLH106* OX lines without stress we speculate that they are involved in abiotic stress.

**Jasmonic Acid Signaling**

Jasmonic acid (JA) is a plant hormone that mediates diverse biological phenomena. JA and its derivatives, collectively referred to as jasmonates, are lipid-derived plant hormones ubiquitous in the plant kingdom. These compounds play pivotal roles in diverse plant biological processes, such as seed maturation, viable pollen production, root growth, tendril coiling, and defense response to biotic and abiotic stresses [31]. Although genes in other groups (*ANAC055* and *CYP94B3*) were also JA responsive, there were eight main JA biosynthesis, responsive, or signaling pathway genes up-regulated in the *bHLH106*-OX lines without stress (S3 Table). These eight genes, *JMT*, *SIB1*, *OPCL1*, *JRG21*, *JAZ8*, *JAZ1*, *JAZ5*, and *AOC3*, were up-regulated more than two-fold, and contain one, two, three, or five G-box core binding motifs in their promoter regions. Transgenic plants over-expressing *JMT*, driven by the *Ubi1* promoter (*Ubi1:AtJMT*), resulted in an increased level of methyl Jasmonate (MeJA) and a reduced grain yield compared with non-transgenic (NT) lines in rice [32]. In addition, ABA levels were increased 1.9 and 1.4 fold in the *Ubi1:AtJMT* transgenic line and drought treated NT line panicles, respectively. The increased ABA content in *Ubi1:JMT* panicles grown in non-drought conditions suggests that MeJA, rather than drought stress, induces ABA biosynthesis under drought conditions [32]. JMT expression was enhanced more than two-fold in *bHLH106* OX lines, and it contains two bHLH106 binding motifs in its promoter.

The *Arabidopsis* *SIGMA FACTOR-BINDING PROTEIN 1* (*SIB1*) gene is rapidly induced by infection with the bacterial pathogen *Pseudomonas syringae*, it might be involved in disease resistance through modulation of salicylate (SA)- and JA-mediated defense responses [33]. Constitutive over-expression of *SIB1* causes plants to hyper-activate defense-related genes following pathogen infection or SA and JA treatments, leading to enhanced resistance to infection by *P. syringae*. *SIB1* was enhanced more than two-fold in *bHLH106* OX lines, and contains five bHLH106 core binding sequences in its promoter. These results suggest that *SIB1* may be regulated by bHLH106, and suggests a role for bHLH106 in JA response.

Terminal reactions of JA biosynthesis in peroxisomes involves reduction of 12-oxo-phytodienoic acid (OPDA) to 3-oxo-2-(2′-pentenyl)-cyclopentane-1-octanoic acid (OPC-8:0), which is subsequently converted to JA by three rounds of beta-oxidation. OPCL1 has a physiological role in activating JA precursors in peroxisomes [34]. *OPCL1* was up-regulated in the *bHLH106* OX lines. Similarly, JAZ8, JAZ1, and AOC3 are involved in the JA signaling pathway [35, 36]. These were induced more than two-fold in the *bHLH106* OX lines, and all contained a G-box motif in their promoter regions. Together, these results demonstrate that bHLH106 regulates important genes involved in the JA signaling pathway.

**ABA Responsive Targets**

The phytohormone ABA regulates plant growth and developmental processes in response to changes in water status. Under water stress, plants increase ABA biosynthesis, triggering responses including stomatal closure to reduce water loss, and expression of genes to produce osmoprotectants. Similar genes are ABA induced in maturing seeds and thought to confer desiccation tolerance to the developing embryo. Although there were many ABA responsive genes present in other groups (e.g., At5g59550, At5g67450, At3g15500, and At5g13330), we grouped five genes into an ABA specific group (S3 Table). All these genes were up-regulated in the transgenic overexpression lines, as they contain G-box motifs in their promoters it indicates that they are direct targets of bHLH106. An ABA induced gene *RING-DUF1117* (*AtRDUF1*) was up-regulated two-fold in the *bHLH106*-OX lines, and has two G-box core sequences in its promoter. Another RING-DUF1117 family gene (*AtRDUF2*) was also present in the zinc finger group (S3 Table), its expression was enhanced more than 10-fold. Both these genes play combinatorial yet distinguishable roles in ABA mediated dehydration stress response [21]. In addition, an unknown protein (At1g05575) was also ABA responsive [37]. To gain further insight into the role of protein tyrosine phosphatase (PTP) in ABA signaling, a T-DNA line of (At3g02800: *AtPFA-DSP3*) was grown, but this did not show any phenotypic differences compared with the wild type. However, T-DNA lines of *phs1* (At5g23720), *ptpkis1* (At3g52180), and *mkp1* (At3g55270) were sensitive to 10 μM ABA [38]. ROP10, a member of the ROP small GTPases is a negative regulator of ABA responses. In deciphering the mechanism of the ROP10-mediated ABA signaling pathway, *BCS1* (At3g50930) was activated in *rop10-1* by 1 mM ABA [37]. *BCS1* was enhanced more than five-fold in over-expression lines of *bHLH106*, and contains one G-box motif in its promoter.

*NUDIX HYDROLASE HOMOLOG 21* (*AtNUDT21*) was enhanced two-fold in the *bHLH106*-OX lines, it contains two core G-box binding sequences in its promoter, at positions 2488 and 2783 (S3 Table). An effort to define Ca^2+^ responsive genes in *Arabidopsis* that contain *cis* elements in their promoters revealed 230 genes [39]. In 162 of the upregulated genes, there was a significant occurrence of two ABA specific cis elements: ABRE, 5’-CACGTG(T/C/G)-3’, and its coupling element ABRE-CE, 5’-(C/A)ACGCG(T/C/A)-3’, in the promoter. *AtNUDT21* was also upregulated, this gene contains multiple ABRE or ABRE-CE motifs in its promoter [39]. These results support our findings that *AtNUDT21* is an ABA responsive gene controlled by bHLH106.

**WRKY Factors**

WRKY transcription factors are one of the largest families of transcriptional regulators in plants. As they act as repressors or activators, it follows that members of the family play roles in both the repression and de-repression of important plant processes. Furthermore, a single WRKY transcription factor may be involved in regulating several different processes. An understanding of the mechanisms underlying signaling and transcriptional regulation revealed that WRKY proteins function via interactions with a diverse array of protein partners, including MAP kinases, MAP kinase kinases, calmodulin, histone deacetylases, resistance proteins, and other WRKY transcription factors [40]. bHLH106 directly regulates three WRKY transcription factors *AtWRKY38*, *AtWRKY45*, and *AtWRKY75*, these have one, two, and three G-box motifs in their promoters, respectively. All three genes were upregulated more than two-fold in the over-expression lines. *WRKY38* and *WRKY45* are involved in defense [41, 42], while *WRKY75* is involved in the nutrient starvation response [43].

**Direct Down-Regulated Genes**

Ninety-eight genes were down-regulated by bHLH106, among these were 36 direct targets containing G-box core sequence in their promoters. Among these, eight genes were involved in salt, cold, or drought stress response, and these genes were down-regulated two-fold or more in the over-expression lines (S4 Table). Expression of two SAUR-like auxin responsive family gene members was reduced in the *bHLH106* OX lines; similarly, their expression was also reduced in *MKK2* over-expression lines [44]. *MKK2* is specifically induced by cold, salt, and stress induced MEKK1, and it directly targets MPK4 and MPK6. In addition to MPK4 and MPK6, MEKK1 also activates MPK3 [11]. *MKK9* was also activated in the bHLH106-OX lines; this gene is upstream to MPK3 and MPK6 [12]. These results demonstrate that bHLH106 regulates a cascade of MKK pathways involved in the regulation of cold and salt stress.

A gene, responsible for encoding an 8S pre-ribosomal assembly protein, was down-regulated approximately four-fold in the *bHLH106*-OX lines (S4 Table); it contains one G-box motif in its promoter. This gene was also down-regulated following 14 days of cold acclimation in *Arabidopsis* [45]. This result supports our findings that bHLH106 regulates important genes involved in salt and cold regulation.

MYB-transcription factor 29 (MYB29) is involved in aliphatic glucosinolate biosynthesis under drought stress [46], and a MYB-like transcription factor (At5g17300) is involved in freezing tolerance in *Arabidopsis* [47]. *MYB29* and the MYB-like transcription factor At5g17300 were both down-regulated two-fold, and contain one and two G-box motifs in their promoters, respectively (S4 Table). These results demonstrate that MYB proteins involved in stress response are regulated by bHLH106.

An ATP-binding microtubule motor family protein (At4g38950) was up-regulated in the wild-type under iron deficiency, but was unchanged in Fe-deficient conditions induced by the *transcription factor 1* (*fit1*) mutant line, therefore indicating its importance in the iron signaling pathway [7]. This gene was down-regulated in the *bHLH106* OX lines, and it has one G-box core sequence in its promoter (S2 Table). This result, in conjunction with the iron transport genes up-regulated in the *bHLH106* over-expression line, indicates that bHLH106 plays a role in regulating iron response genes.

**References**

1. Davletova S, Rizhsky L, Liang H, Shengqiang Z, Oliver DJ, Coutu J, et al. Cytosolic ascorbate peroxidase 1 is a central component of the reactive oxygen gene network of *Arabidopsis*. Plant Cell. 2005;17: 268-281. doi: 10.1105/tpc.104.026971

2. Zhao J, Barkla BJ, Marshall J, Pittman JK, Hirschi KD. The *Arabidopsis cax3* mutants display altered salt tolerance, pH sensitivity and reduced plasma membrane H^+^-ATPase activity. Planta. 2008;227: 659-669. doi: 10.1007/s00425-007-0648-2

3. Medina J, Rodriguez-Franco M, Penalosa A, Carrascosa MJ, Neuhaus G, Salinas J. *Arabidopsis* mutants deregulated in *RCI2A* expression reveal new signaling pathways in abiotic stress responses. Plant J. 2005;42: 586-597. doi: 10.1111/j.1365-313X.2005.02400.x

4. Kang HG, Kim J, Kim B, Jeong H, Choi SH, Kim EK, et al. Overexpression of FTL1/DDF1, an AP2 transcription factor, enhances tolerance to cold, drought, and heat stresses in *Arabidopsis thaliana*. Plant Sci. 2011;180: 634-641. doi: 10.1016/j.plantsci.2011.01.002

5. Sharabi-Schwager M, Lers A, Samach A, Guy CL, Porat R. Overexpression of the CBF2 transcriptional activator in *Arabidopsis* delays leaf senescence and extends plant longevity. J Exp Bot. 2010;61: 261-273. doi: 10.1093/jxb/erp300

6. Brumbarova T, Bauer P. Iron-mediated control of the basic helix-loop-helix protein FER, a regulator of iron uptake in tomato. Plant Physiol. 2005;137: 1018-1026. doi: 10.1104/pp.104.054270

7. Colangelo EP, Guerinot ML. The essential basic helix-loop-helix protein FIT1 is required for the iron deficiency response. Plant Cell. 2004;16: 3400-3412. doi: 10.1105/tpc.104.024315

8. Long TA, Tsukagoshi H, Busch W, Lahner B, Salt DE, Benfey PN. The bHLH transcription factor POPEYE regulates response to iron deficiency in *Arabidopsis* roots. Plant Cell. 2010;22: 2219-2236. doi: 10.1105/tpc.110.074096

9. Korshunova YO, Eide D, Clark WG, Guerinot ML, Pakrasi HB. The IRT1 protein from *Arabidopsis thaliana* is a metal transporter with a broad substrate range. Plant Mol Biol. 1999;40: 37-44.

10. Henriques R, Jasik J, Klein M, Martinoia E, Feller U, Schell J, et al. Knock-out of *Arabidopsis* metal transporter gene IRT1 results in iron deficiency accompanied by cell differentiation defects. Plant Mol Biol. 2002;50: 587-597.

11. Conroy C, Ching J, Gao Y, Wang X, Rampitsch C, Xing T. Knockout of AtMKK1 enhances salt tolerance and modifies metabolic activities in *Arabidopsis*. Plant Signal Behav. 2013;8: e24206. doi: 10.4161/psb.24206

12. Xu J, Li Y, Wang Y, Liu H, Lei L, Yang H, et al. Activation of MAPK kinase 9 induces ethylene and camalexin biosynthesis and enhances sensitivity to salt stress in *Arabidopsis*. J Biol Chem. 2008;283: 26996-27006. doi: 10.1074/jbc.M801392200

13. Eschen-Lippold L, Bethke G, Palm-Forster MA, Pecher P, Bauer N, Glazebrook J, et al. MPK11-a fourth elicitor-responsive mitogen-activated protein kinase in *Arabidopsis thaliana*. Plant Signal Behav. 2012;7: 1203-1205. doi: 10.4161/psb.21323

14. Suzuki N, Rizhsky L, Liang H, Shuman J, Shulaev V, Mittler R. Enhanced tolerance to environmental stress in transgenic plants expressing the transcriptional coactivator multiprotein bridging factor 1c. Plant Physiol. 2005;139: 1313-1322. doi: 10.1104/pp.105.070110

15. Mizoguchi T, Hayashida N, Yamaguchi-Shinozaki K, Kamada H, Shinozaki K. Two genes that encode ribosomal-protein S6 kinase homologs are induced by cold or salinity stress in *Arabidopsis thaliana*. FEBS Lett. 1995;358: 199-204.

16. Bu Q, Jiang H, Li CB, Zhai Q, Zhang J, Wu X, et al. Role of the *Arabidopsis thaliana* NAC transcription factors ANAC019 and ANAC055 in regulating jasmonic acid-signaled defense responses. Cell Res. 2008;18: 756-567. doi: 10.1038/cr.2008.53

17. Abe H, Urao T, Ito T, Seki M, Shinozaki K, Yamaguchi-Shinozaki K. *Arabidopsis* AtMYC2 (bHLH) and AtMYB2 (MYB) function as transcriptional activators in abscisic acid signaling. Plant Cell. 2003;15: 63-78.

18. Balazadeh S, Wu A, Mueller-Roeber B. Salt-triggered expression of the ANAC092-dependent senescence regulon in *Arabidopsis thaliana*. Plant Signal Behav. 2010;5: 733-735.

19. Stroher E, Wang XJ, Roloff N, Klein P, Husemann A, Dietz KJ. Redox-dependent regulation of the stress-induced zinc-finger protein SAP12 in *Arabidopsis thaliana*. Mol Plant. 2009;2: 357-367. doi: 10.1093/mp/ssn084

20. Ma S, Gong Q, Bohnert HJ. An *Arabidopsis* gene network based on the graphical Gaussian model. Genome Res. 2007;17: 1614-1625. doi: 10.1101/gr.6911207

21. Kim SJ, Ryu MY, Kim WT. Suppression of *Arabidopsis* RING-DUF1117 E3 ubiquitin ligases, AtRDUF1 and AtRDUF2, reduces tolerance to ABA-mediated drought stress. Biochem Biophys Res Commun. 2012;420: 141-147. doi: 10.1016/j.bbrc.2012.02.131

22. Huang S, Spielmeyer W, Lagudah ES, Munns R. Comparative mapping of HKT genes in wheat, barley, and rice, key determinants of Na^+^ transport, and salt tolerance. J Exp Bot. 2008;59: 927-937. doi: 10.1093/jxb/ern033

23. Soitamo AJ, Piippo M, Allahverdiyeva Y, Battchikova N, Aro EM. Light has a specific role in modulating *Arabidopsis* gene expression at low temperature. BMC Plant Biol. 2008;8: 13. doi: 10.1186/1471-2229-8-13

24. Mehterov N, Balazadeh S, Hille J, Toneva V, Mueller-Roeber B, Gechev T. Oxidative stress provokes distinct transcriptional responses in the stress-tolerant atr7 and stress-sensitive loh2 *Arabidopsis thaliana* mutants as revealed by multi-parallel quantitative real-time PCR analysis of ROS marker and antioxidant genes. Plant Physiol Biochem. 2012;59: 20-29. doi: 10.1016/j.plaphy.2012.05.024

25. Lee SJ, Park JH, Lee MH, Yu JH, Kim SY. Isolation and functional characterization of CE1 binding proteins. BMC Plant Biol. 2010;10: 277. doi: 10.1186/1471-2229-10-277

26. Kreps JA, Wu Y, Chang HS, Zhu T, Wang X, Harper JF. Transcriptome changes for *Arabidopsis* in response to salt, osmotic, and cold stress. Plant Physiol. 2002;130: 2129-2141. doi: 10.1104/pp.008532

27. Umezawa T, Okamoto M, Kushiro T, Nambara E, Oono Y, Seki M, et al. CYP707A3, a major ABA 8'-hydroxylase involved in dehydration and rehydration response in *Arabidopsis thaliana*. Plant J. 2006;46: 171-182. doi: 10.1111/j.1365-313X.2006.02683.x

28. Heitz T, Widemann E, Lugan R, Miesch L, Ullmann P, Desaubry L, et al. Cytochromes P450 CYP94C1 and CYP94B3 catalyze two successive oxidation steps of plant hormone Jasmonoyl-isoleucine for catabolic turnover. J Biol Chem. 2012;287: 6296-6306. doi: 10.1074/jbc.M111.316364

29. Kandel S, Sauveplane V, Compagnon V, Franke R, Millet Y, Schreiber L, et al. Characterization of a methyl jasmonate and wounding-responsive cytochrome P450 of *Arabidopsis thaliana* catalyzing dicarboxylic fatty acid formation in vitro. FEBS J. 2007;274: 5116-5127. doi: 10.1111/j.1742-4658.2007.06032.x

30. Schweighofer A, Hirt H, Meskiene I. Plant PP2C phosphatases: Emerging functions in stress signaling. Trends Plant Sci. 2004;9: 236-243. doi: 10.1016/j.tplants.2004.03.007

31. Creelman RA, Mullet JE. Jasmonic acid distribution and action in plants: Regulation during development and response to biotic and abiotic stress. Proc Natl Acad Sci U S A. 1995;92: 4114-4119.

32. Kim EH, Kim YS, Park SH, Koo YJ, Choi YD, Chung YY, et al. Methyl jasmonate reduces grain yield by mediating stress signals to alter spikelet development in rice. Plant Physiol. 2009;149: 1751-1760. doi: 10.1104/pp.108.134684

33. Xie YD, Li W, Guo D, Dong J, Zhang Q, Fu Y, et al. The *Arabidopsis* gene SIGMA FACTOR-BINDING PROTEIN 1 plays a role in the salicylate- and jasmonate-mediated defence responses. Plant Cell Environ. 2010;33: 828-839. doi: 10.1111/j.1365-3040.2009.02109.x

34. Koo AJ, Chung HS, Kobayashi Y, Howe GA. Identification of a peroxisomal acyl-activating enzyme involved in the biosynthesis of jasmonic acid in *Arabidopsis*. J Biol Chem. 2006;281: 33511-33520. doi: 10.1074/jbc.M607854200

35. Shyu C, Figueroa P, Depew CL, Cooke TF, Sheard LB, Moreno JE, et al. JAZ8 lacks a canonical degron and has an EAR motif that mediates transcriptional repression of jasmonate responses in *Arabidopsis*. Plant Cell. 2012;24: 536-550. doi: 10.1105/tpc.111.093005

36. Stenzel I, Ischebeck T, Quint M, Heilmann I. Variable regions of PI4P 5-kinases direct PtdIns(4,5)P(2) toward alternative regulatory functions in tobacco pollen tubes. Front Plant Sci. 2011;2: 114. doi: 10.3389/fpls.2011.00114

37. Xin Z, Zhao Y, Zheng ZL. Transcriptome analysis reveals specific modulation of abscisic acid signaling by ROP10 small GTPase in *Arabidopsis*. Plant Physiol. 2005;139: 1350-1365. doi: 10.1104/pp.105.068064

38. Quettier AL, Bertrand C, Habricot Y, Miginiac E, Agnes C, Jeannette E, et al. The phs1-3 mutation in a putative dual-specificity protein tyrosine phosphatase gene provokes hypersensitive responses to abscisic acid in *Arabidopsis thaliana*. Plant J. 2006;47: 711-719. doi: 10.1111/j.1365-313X.2006.02823.x

39. Kaplan B, Davydov O, Knight H, Galon Y, Knight MR, Fluhr R, et al. Rapid transcriptome changes induced by cytosolic Ca^2+^ transients reveal ABRE-related sequences as Ca^2+^-responsive cis elements in *Arabidopsis*. Plant Cell. 2006;18: 2733-2748. doi: 10.1105/tpc.106.042713

40. Rushton PJ, Somssich IE, Ringler P, Shen QJ. WRKY transcription factors. Trends Plant Sci. 2010;15: 247-258. doi: 10.1016/j.tplants.2010.02.006

41. Kim KC, Lai Z, Fan B, Chen Z. *Arabidopsis* WRKY38 and WRKY62 transcription factors interact with histone deacetylase 19 in basal defense. Plant Cell. 2008;20: 2357-2371. doi: 10.1105/tpc.107.055566

42. Shimono M, Sugano S, Nakayama A, Jiang CJ, Ono K, Toki S, et al. Rice WRKY45 plays a crucial role in benzothiadiazole-inducible blast resistance. Plant Cell. 2007;19: 2064-2076. doi: 10.1105/tpc.106.046250

43. Devaiah BN, Karthikeyan AS, Raghothama KG. WRKY75 transcription factor is a modulator of phosphate acquisition and root development in *Arabidopsis*. Plant Physiol. 2007;143: 1789-1801. doi: 10.1104/pp.106.093971

44. Teige M, Scheikl E, Eulgem T, Doczi R, Ichimura K, Shinozaki K, et al. The MKK2 pathway mediates cold and salt stress signaling in *Arabidopsis*. Mol Cell. 2004;15: 141-152. doi: 10.1016/j.molcel.2004.06.023

45. Hannah MA, Heyer AG, Hincha DK. A global survey of gene regulation during cold acclimation in *Arabidopsis thaliana*. PLoS Genet. 2005;1: e26. doi: 10.1371/journal.pgen.0010026

46. Mewes HW, Frishman D, Guldener U, Mannhaupt G, Mayer K, Mokrejs M, et al. MIPS: A database for genomes and protein sequences. Nucleic Acids Res. 2002;30: 31-34

47. Meissner M, Orsini E, Ruschhaupt M, Melchinger AE, Hincha DK, Heyer AG. Mapping quantitative trait loci for freezing tolerance in a recombinant inbred line population of *Arabidopsis thaliana* accessions Tenela and C24 reveals REVEILLE1 as negative regulator of cold acclimation. Plant Cell Environ. 2012. doi: 10.1111/pce.12054
