# Supplementary figures and images for: bHLH106 Integrates Functions of Multiple Genes through Their G-Box to Confer Salt Tolerance on Arabidopsis
Source: PLoS One. 2015 May 15;10(5):e0126872. doi: 10.1371/journal.pone.0126872 (PMC4433118; doi:10.1371/journal.pone.0126872)

## A phenotypes under abiotic stresses

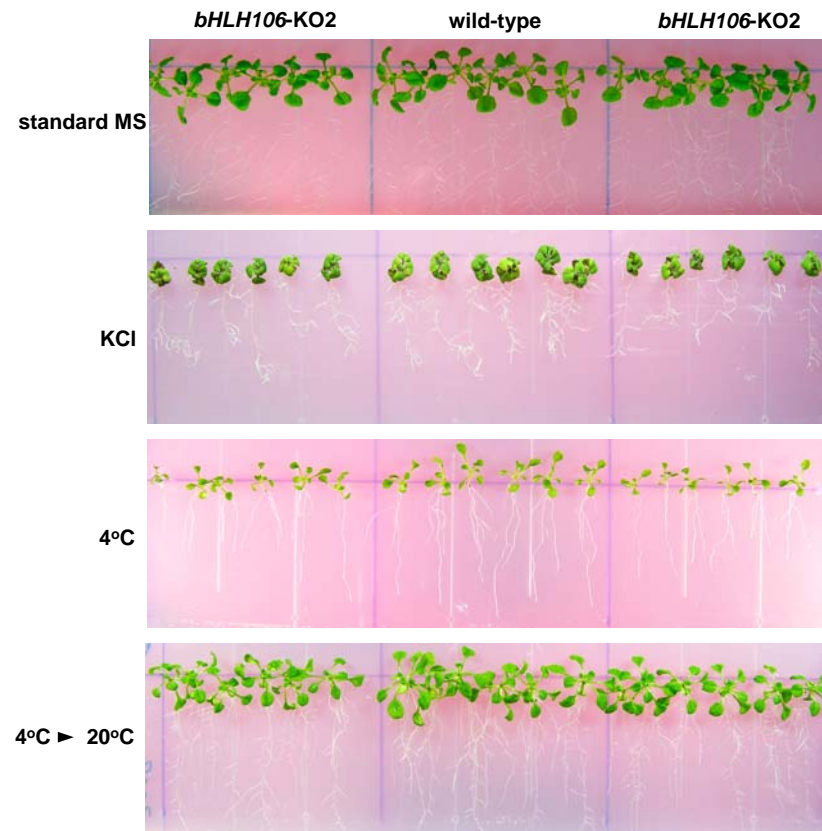

## B statistic data of root length

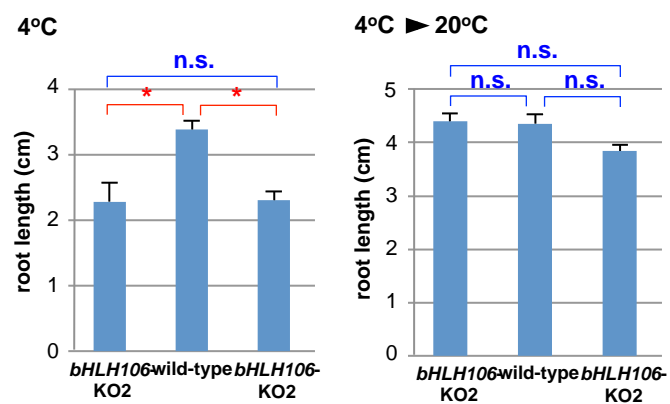

Figure S1

Supplement: S1 Fig — (A) bHLH106-KO2 and wild-type plants were germinated and grown on standard MS medium for 5 days, and then transferred to medium with or without 110 mM KCl. Plants were incubated at 4°C for 2 weeks after transferring to standard MS in rectangular culture plates (4°C), and returned to the culture condition at 20°C for 1 week (4°C > 20°C). (B) Statistic data of root length in the same experiments shown in panel A. Error bars represent ±SEM from six experimental replicates. Here are “n.s.” for no significant difference and * for P < 0.05 in ANOVA. (PDF) [file pone.0126872.s002.pdf]

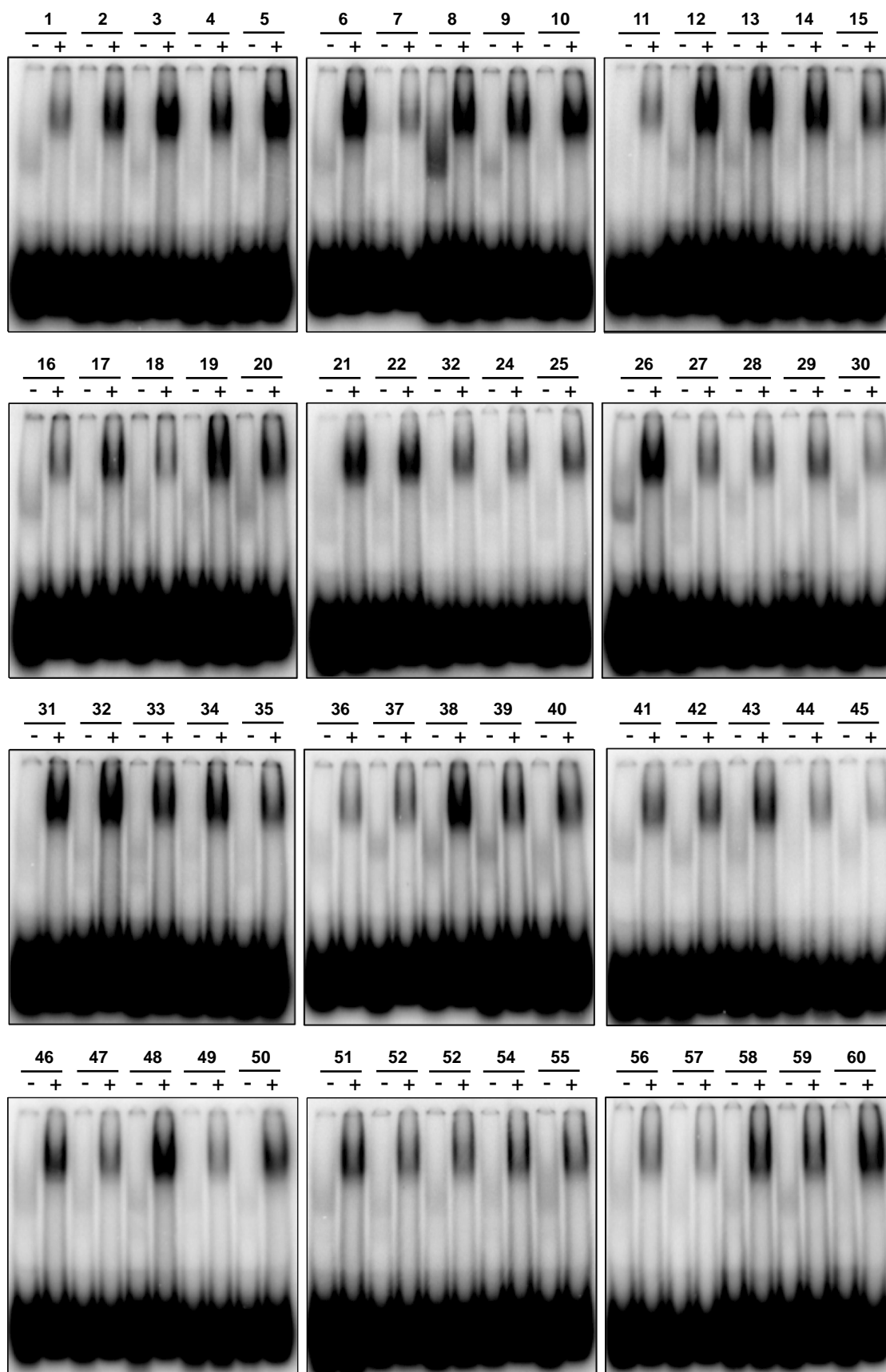

Figure S3

Supplement: S3 Fig — The locus number of these genes are given in S5 Table. (PDF) [file pone.0126872.s004.pdf]

## A hierarchical clustering

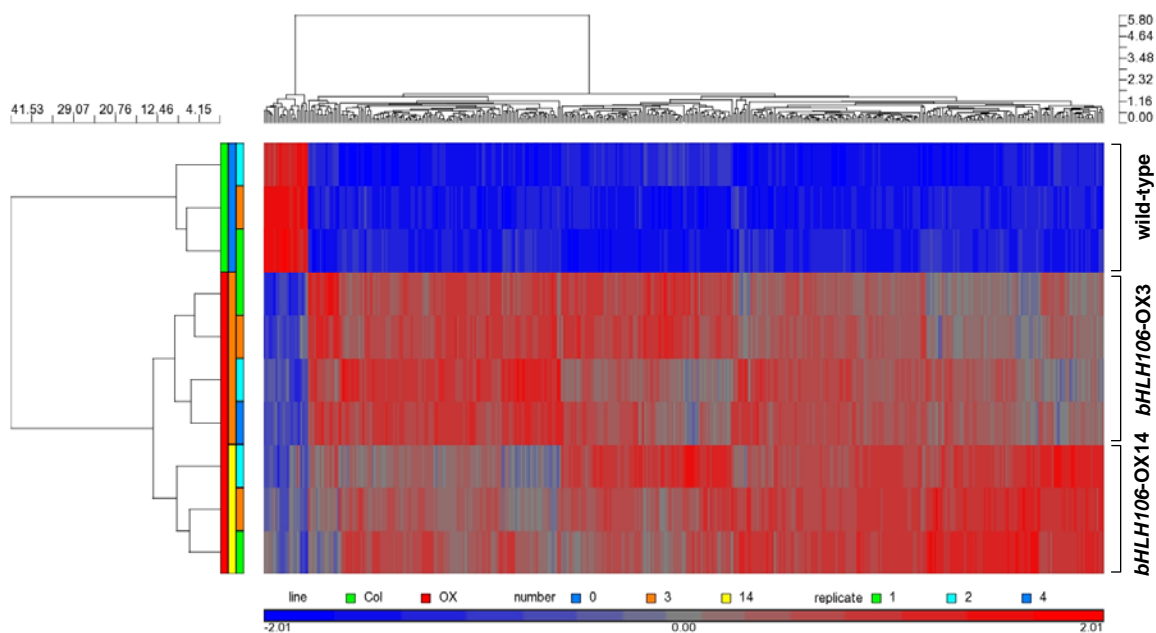

## B volcano plot

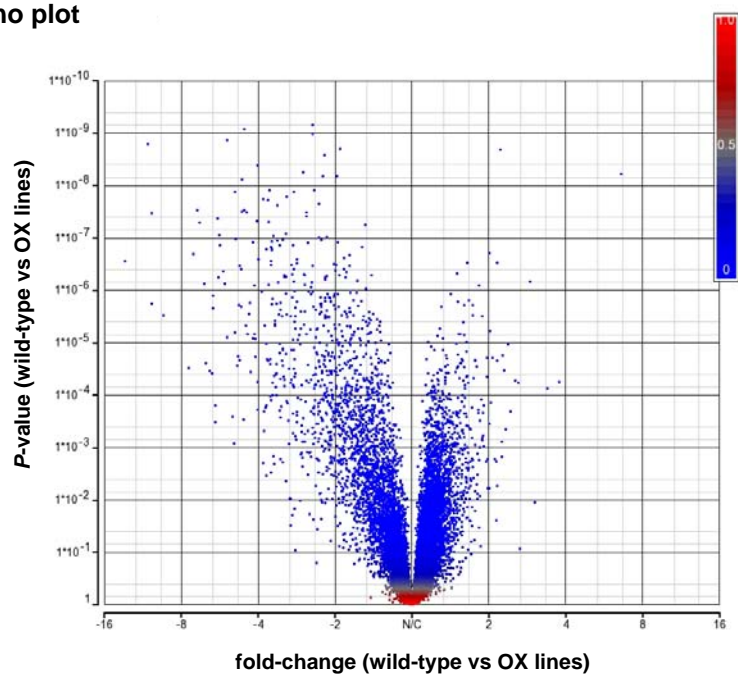

Figure S4

Supplement: S4 Fig — (A) Hierarchical clustering. The analyses were done for gene expression in the lines bHLH106-OX3, bHLH106-OX14, and wild-type in their triplicate or quadruplicate by Partek Genomics Suit 6.6. (B) Volcano plot. The analysis was performed as described for Panel A. (PDF) [file pone.0126872.s005.pdf]
