# Supplementary material for: bHLH106 Integrates Functions of Multiple Genes through Their G-Box to Confer Salt Tolerance on Arabidopsis
Source: PLoS One. 2015 May 15;10(5):e0126872. doi: 10.1371/journal.pone.0126872 (PMC4433118; doi:10.1371/journal.pone.0126872)
Supplement: S2 Fig — (A) Expression of bHLH106 in leaves of the rescue (RES) lines. bHLH106-KO1 lines was transformed with a construct for bHLH106 overexpression (OX) driven by the CaVM 35S promoter. Homozygous lines F2 were for analysis. Expression was determined by real-time RT-PCR and normalized using ACTIN2 (ACT2). Error bars represent ±SEM from three experimental replicates. All the P-values are less than 0.01 between the wild-type and the rescue lines. (B) Phenotypes of bHLH106-RES lines grown on culture medium containing 125 mM NaCl. The culture and treatment of plants with NaCl was performed as described in the legend for Fig 3. (C) Statistic data of leaf extent in the same experiments shown in panel B. Error bars represent ±SEM from six experimental replicates. Here is * for P < 0.05 in ANOVA. (PDF) [file pone.0126872.s003.pdf]

A transcripts for *bHLH106* in its rescue lines

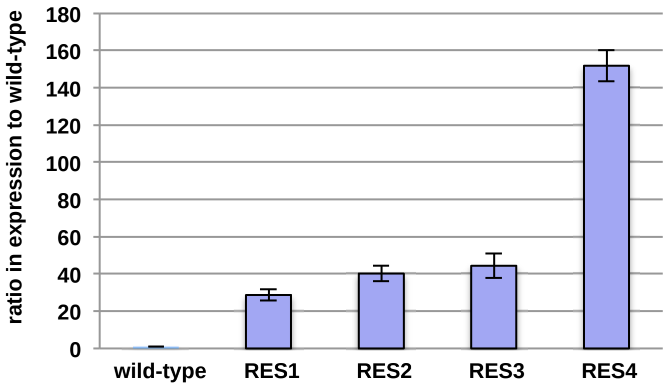

B phenotypes on salt-containing medium

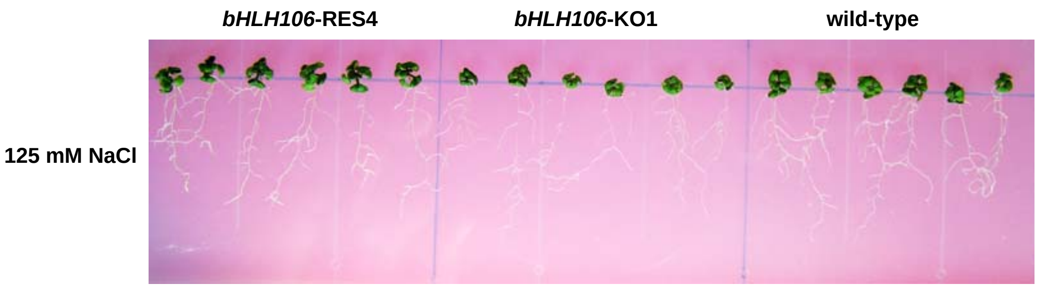

C statistical data of leaf extent

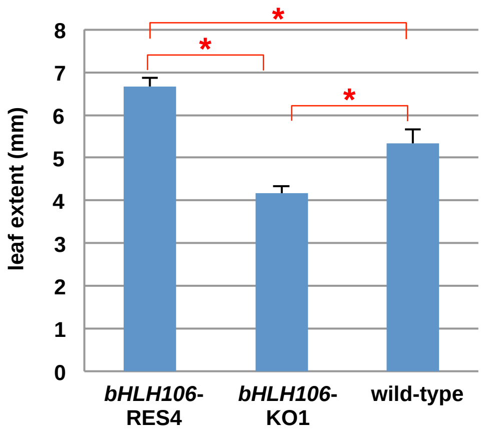

Figure S2
