# Supplementary material for: bHLH106 Integrates Functions of Multiple Genes through Their G-Box to Confer Salt Tolerance on Arabidopsis
Source: PLoS One. 2015 May 15;10(5):e0126872. doi: 10.1371/journal.pone.0126872 (PMC4433118; doi:10.1371/journal.pone.0126872)
Supplement: S1 Table — (DOCX) [file pone.0126872.s006.docx]

**Table S1.** Genes satisfying both criteria of presence of G-box in promoters and up-regulation in OX Lines ^a^

| Probe ^b^ | Atg Number | Log ^c^  Ratio | G-Box | Position ^d^ | Annotation |
| --- | --- | --- | --- | --- | --- |
| 247708_at | AT5G59550 | 4.2 | 1 | 2979 | zinc finger (C3HC4-type RING finger) family protein |
| 255532_at | AT4G02170 | 3.4 | 2 | 521, 532 | similar to unknown protein |
| 261157_at | AT1G34510 | 3.4 | 1 | 1490 | peroxidase, putative |
| 251625_at | AT3G57260 | 3.4 | 1 | 2762 | BGL2 (PATHOGENESIS-RELATED PROTEIN 2); |
| 260568_at | AT2G43570 | 3.3 | 1 | 1277 | chitinase, putative |
| 266070_at | AT2G18660 | 3.0 | 1 | 793 | EXLB3 (EXPANSIN-LIKE B3 PRECURSOR) |
| 253628_at | AT4G30280 | 3.0 | 1 | 769 | ATXTH18/XTH18 |
| 252131_at | AT3G50930 | 2.9 | 1 | 209 | AAA-type ATPase family protein |
| 253998_at | AT4G26010 | 2.8 | 1 | 1079 | peroxidase, putative |
| 247208_at | AT5G64870 | 2.7 | 2 | 2232 | similar to unknown protein |
| 267140_at | AT2G38250 | 2.6 | 3 | 950, 1790, 1875 | DNA-binding protein-related |
| 263935_at | AT2G35930 | 2.6 | 2 | 2339, 2727 | U-box domain-containing protein |
| 247729_at | AT5G59530 | 2.6 | 1 | 1382 | 2-oxoglutarate-dependent dioxygenase, putative |
| 253259_at | AT4G34410 | 2.5 | 4 | 993, 1830, 2581, 2867 | AP2 domain-containing transcription factor, putative |
| 256252_at | AT3G11340 | 2.5 | 1 | 1130 | UDP-glucoronosyl/UDP-glucosyl transferase family protein |
| 264758_at | AT1G61340 | 2.4 | 1 | 2759 | F-box family protein |
| 266246_at | AT2G27690 | 2.4 | 1 | 947 | CYP94C1 |
| 250778_at | AT5G05500 | 2.4 | 1 | 1670 | pollen Ole e 1 allergen and extensin family protein |
| 254314_at | AT4G22470 | 2.3 | 1 | 2686 | protease inhibitor/seed storage/lipid transfer protein |
| 264434_at | AT1G10340 | 2.2 | 2 | 450, 1103 | ankyrin repeat family protein |
| 264005_at | AT2G22470 | 2.2 | 2 | 2702, 2751 | AGP2 (ARABINOGALACTAN-PROTEIN 2) |
| 251774_at | AT3G55840 | 2.2 | 1 | 346 | similar to unknown protein |
| 248178_at | AT5G54370 | 2.2 | 1 | 2311 | late embryogenesis abundant protein-related / LEA protein-related |
| 250445_at | AT5G10760 | 2.1 | 3 | 553, 573, 1619 | aspartyl protease family protein |
| 262045_at | AT1G80240 | 2.1 | 2 | 1924, 2208 | similar to unknown protein |
| 261470_at | AT1G28370 | 2.1 | 1 | 2658 | ATERF11/ERF11 (ERF domain protein 11) |
| 260230_at | AT1G74500 | 2.1 | 1 | 1779 | bHLH family protein |
| 266821_at | AT2G44840 | 2.1 | 1 | 1985 | ATERF13/EREBP |
| 256017_at | AT1G19180 | 2.0 | 3 | 149, 1840, 2936 | JAZ1/TIFY10A (JASMONATE-ZIM-DOMAIN PROTEIN 1) |
| 263539_at | AT2G24850 | 2.0 | 1 | 1306 | TAT3 (TYROSINE AMINOTRANSFERASE 3); |
| 254550_at | AT4G19690 | 1.9 | 1 | 2301 | IRT1 (IRON-REGULATED TRANSPORTER 1) |
| 255941_at | AT1G20350 | 1.8 | 2 | 28, 806 | ATTIM17-1 |
| 258682_at | AT3G08720 | 1.8 | 2 | 1779, 2916 | ATPK19 (ARABIDOPSIS THALIANA PROTEIN KINASE 19); kinase |
| 263182_at | AT1G05575 | 1.8 | 1 | 1807 | unknown protein |
| 264648_at | AT1G09080 | 1.8 | 1 | 2816 | BIP3; ATP binding |
| 260744_at | AT1G15010 | 1.8 | 1 | 1589 | similar to unknown protein |
| 261443_at | AT1G28480 | 1.8 | 1 | 1883 | GRX480; thiol-disulfide exchange intermediate |
| 259276_at | AT3G01190 | 1.8 | 1 | 1028 | peroxidase 27 (PER27) (P27) (PRXR7) |
| 254767_s_at | AT4G13290 | 1.8 | 1 | 2446 | CYP71A19 |
| 245329_at | AT4G14365 | 1.8 | 1 | 85 | zinc finger (C3HC4-type RING finger) family protein |
| 253044_at | AT4G37290 | 1.8 | 1 | 2223 | similar to unknown protein |
| 252833_at | AT4G40090 | 1.8 | 1 | 946 | AGP3 (ARABINOGALACTAN-PROTEIN 3) |
| 248164_at | AT5G54490 | 1.8 | 1 | 2449 | PBP1 (PINOID-BINDING PROTEIN 1); calcium ion binding |
| 246302_at | AT3G51860 | 1.7 | 3 | 1057, 1654, 2302 | CAX3 (cation exchanger 3); cation:cation antiporter |
| 249983_at | AT5G18470 | 1.7 | 3 | 1462, 2293, 2303 | curculin-like (mannose-binding) lectin family protein |
| 255937_at | AT1G12610 | 1.7 | 2 | 1750, 1792 | DDF1 (DWARF AND DELAYED FLOWERING 1) |
| 248964_at | AT5G45340 | 1.7 | 2 | 2098, 2858 | CYP707A3 |
| 261033_at | AT1G17380 | 1.7 | 1 | 2113 | JAZ5/TIFY11A (JASMONATE-ZIM-DOMAIN PROTEIN 5) |
| 262085_at | AT1G56060 | 1.7 | 1 | 1628 | similar to unknown protein |
| 265597_at | AT2G20142 | 1.7 | 1 | 1415 | transmembrane receptor |
| 249094_at | AT5G43890 | 1.7 | 1 | 1358 | SUPER1/YUCCA5 (SUPPRESSOR OF ER1) |
| 247755_at | AT5G59090 | 1.7 | 1 | 267 | ATSBT4.12; subtilase |
| 263216_s_at | AT1G30730 | 1.6 | 2 | 595, 630 | FAD-binding domain-containing protein |
| 259428_at | AT1G01560 | 1.6 | 1 | 1413 | ATMPK11 (Arabidopsis thaliana MAP kinase 11) |
| 262666_at | AT1G14080 | 1.6 | 1 | 45 | FUT6 (fucosyltransferase 6) |
| 261648_at | AT1G27730 | 1.6 | 1 | 2760 | STZ (SALT TOLERANCE ZINC FINGER) |
| 263295_at | AT2G14210 | 1.6 | 1 | 2665 | ANR1; DNA binding / transcription factor |
| 258792_at | AT3G04640 | 1.6 | 1 | 2383 | glycine-rich protein |
| 256576_at | AT3G28210 | 1.6 | 1 | 947 | PMZ; zinc ion binding |
| 255630_at | AT4G00700 | 1.6 | 1 | 2752 | C2 domain-containing protein |
| 246888_at | AT5G26270 | 1.6 | 1 | 1069 | unknown protein |
| 249467_at | AT5G39610 | 1.6 | 1 | 1764 | ANAC092/ATNAC2/ATNAC6 |
| 247071_at | AT5G66640 | 1.6 | 1 | 2035 | LIM domain-containing protein-related |
| 256356_s_at | At1g66500 | 1.6 | 1 | 2915 | zinc finger (C2H2-type) family protein] |
| 261930_at | AT1G22440 | 1.5 | 2 | 745, 2672 | alcohol dehydrogenase, putative |
| 249812_at | AT5G23830 | 1.5 | 2 | 363, 2817 | MD-2-related lipid recognition domain-containing protein |
| 252368_at | AT3G48520 | 1.5 | 1 | 1769 | CYP94B3 |
| 251942_at | AT3G53480 | 1.5 | 1 | 370 | ATPDR9/PDR9 (PLEIOTROPIC DRUG RESISTANCE 9) |
| 251633_at | AT3G57460 | 1.5 | 1 | 2739 | catalytic/ metal ion binding / metalloendopeptidase/ |
| 245250_at | AT4G17490 | 1.5 | 1 | 105 | ATERF6 (ETHYLENE RESPONSIVE ELEMENT BINDING FACTOR 6) |
| 253859_at | AT4G27657 | 1.5 | 1 | 2874 | similar to unknown protein |
| 246993_at | AT5G67450 | 1.5 | 1 | 1995 | AZF1 (ARABIDOPSIS ZINC-FINGER PROTEIN 1) |
| 253827_at | AT4G28085 | 1.4 | 2 | 2213, 2297 | unknown protein |
| 246252_s_at | AT4G37070 | 1.4 | 2 | 2183, 2459 | PLA IVA/PLP1; (Patatin-like protein 1) nutrient reservoir |
| 262373_at | AT1G73120 | 1.4 | 1 | 2806 | similar to hypothetical protein |
| 265439_at | AT2G21045 | 1.4 | 1 | 1537 | similar to unknown protein |
| 267337_at | AT2G39980 | 1.4 | 1 | 1787 | transferase family protein |
| 259040_at | AT3G09270 | 1.4 | 1 | 722 | ATGSTU8 (Arabidopsis thaliana Glutathione S-transferase |
| 257623_at | AT3G26210 | 1.4 | 1 | 2307 | CYP71B23 (cytochrome P450, family Protein) |
| 252679_at | AT3G44260 | 1.4 | 1 | 1422 | CCR4-NOT transcription complex protein, putative |
| 246252_s_at | AT4G37060 | 1.4 | 1 | 2235 | PLA IVB/PLP5 (Patatin-like protein 5) |
| 245944_at | AT5G19520 | 1.4 | 1 | 2864 | mechanosensitive ion channel domain-containing protein |
| 249417_at | AT5G39670 | 1.4 | 1 | 215 | calcium-binding EF hand family protein |
| 247137_at | AT5G66210 | 1.4 | 1 | 2352 | CPK28 (calcium-dependent protein kinase 28) |
| 254767_s_at | AT4G13310 | 1.4 | NA |  | CYP71A20 |
| 245252_at | AT4G17500 | 1.3 | 4 | 434, 955, 2344, 2793 | ATERF-1 (ETHYLENE RESPONSIVE ELEMENT BINDING FACTOR 1) |
| 261135_at | AT1G19610 | 1.3 | 2 | 2158, 2286 | LCR78/PDF1.4 (Low-molecular-weight cysteine-rich 78) |
| 260804_at | AT1G78410 | 1.3 | 2 | 2149, 2198 | VQ motif-containing protein |
| 256647_at | AT3G13610 | 1.3 | 2 | 1321, 2429 | oxidoreductase, 2OG-Fe(II) oxygenase family protein |
| 253104_at | AT4G36010 | 1.3 | 2 | 319, 2931 | pathogenesis-related thaumatin family protein |
| 249454_at | AT5G39520 | 1.3 | 2 | 784, 2888 | similar to unknown protein |
| 259410_at | AT1G13340 | 1.3 | 1 | 1787 | similar to unknown protein |
| 264680_at | AT1G65510 | 1.3 | 1 | 1301 | similar to unknown protein |
| 266834_s_at | AT2G05050 | 1.3 | 1 | 2853 | pseudogene, protein phosphatase 2C |
| 265723_at | AT2G32140 | 1.3 | 1 | 1869 | transmembrane receptor |
| 266993_at | AT2G39210 | 1.3 | 1 | 1647 | nodulin family protein |
| 266834_s_at | AT3G27140 | 1.3 | 1 | 2853 | protein phosphatase 2C, putative |
| 252549_at | AT3G45860 | 1.3 | 1 | 1755 | receptor-like protein kinase, putative |
| 251770_at | AT3G55970 | 1.3 | 1 | 1170 | oxidoreductase, 2OG-Fe(II) oxygenase family protein |
| 266834_s_at | AT4G08260 | 1.3 | 1 | 2853 | protein phosphatase 2C, putative |
| 254784_at | AT4G12720 | 1.3 | 1 | 164 | AtNUDT7 |
| 254158_at | AT4G24380 | 1.3 | 1 | 11 | hydrolase, acting on ester bonds |
| 254075_at | AT4G25470 | 1.3 | 1 | 2884 | CBF2 (FREEZING TOLERANCE QTL 4) |
| 250157_at | AT5G15180 | 1.3 | 1 | 2144 | peroxidase, putative |
| 260950_s_at | AT1G06120 | 1.3 |  |  | fatty acid desaturase family protein |
| 266834_s_at | AT2G30020 | 1.3 |  |  | protein phosphatase 2C, putative |
| 261526_at | AT1G14370 | 1.2 | 2 | 620, 1404 | APK2A (PROTEIN KINASE 2A); kinase |
| 261150_at | AT1G19640 | 1.2 | 2 | 730, 794 | JMT (JASMONIC ACID CARBOXYL METHYLTRANSFERASE |
| 246600_at | AT5G14930 | 1.2 | 2 | 2008, 2094 | SAG101 (SENESCENCE-ASSOCIATED GENE 101) |
| 249754_at | AT5G24530 | 1.2 | 2 | 756, 817 | oxidoreductase, 2OG-Fe(II) oxygenase family protein |
| 264580_at | AT1G05340 | 1.2 | 1 | 2896 | similar to unknown protein |
| 265160_at | AT1G31050 | 1.2 | 1 | 2409 | transcription factor |
| 266545_at | AT2G35290 | 1.2 | 1 | 1563 | similar to unnamed protein product |
| 256627_at | AT3G19970 | 1.2 | 1 | 1880 | similar to unknown protein |
| 258080_at | AT3G25930 | 1.2 | 1 | 222 | universal stress protein (USP) family protein |
| 258068_at | AT3G25990 | 1.2 | 1 | 365 | DNA-binding protein GT-1-related |
| 252421_at | AT3G47540 | 1.2 | 1 | 2847 | chitinase, putative |
| 251745_at | AT3G55980 | 1.2 | 1 | 2496 | zinc finger (CCCH-type) family protein |
| 255524_at | AT4G02330 | 1.2 | 1 | 23 | ATPMEPCRB; pectinesterase |
| 254707_at | AT4G18010 | 1.2 | 1 | 2884 | IP5PII (INOSITOL POLYPHOSPHATE 5-PHOSPHATASE II) |
| 254361_at | AT4G22212 | 1.2 | 1 | 141 | Encodes a defensin-like (DEFL) family protein. |
| 254256_at | AT4G23180 | 1.2 | 1 | 1202 | CRK10 (CYSTEINE-RICH RLK10); kinase |
| 253268_s_at | AT4G34135 | 1.2 | 1 | 28 | UGT73B2; UDP-glucosyltransferase/UDP-glycosyltransferase |
| 249890_at | AT5G22570 | 1.2 | 1 | 1166 | WRKY38 (WRKY DNA-binding protein 38); transcription factor |
| 246927_s_at | AT5G25250 | 1.2 | 1 | 526 | similar to unknown protein |
| 247493_at | AT5G61900 | 1.2 | 1 | 2475 | BON1 (BONZAI1); calcium-dependent phospholipid binding |
| 267230_at | At2g44080 | 1.2 |  |  | ARL (ARGOS-LIKE) |
| 246293_at | AT3G56710 | 1.1 | 5 | 567, 1193, 1539, 1863, 2677 | SIB1 (SIGMA FACTOR BINDING PROTEIN 1); binding |
| 251026_at | AT5G02200 | 1.1 | 4 | 1594, 2296, 2777, 2831 | FHL (FAR-RED-ELONGATED HYPOCOTYL1-LIKE) protein binding |
| 245976_at | AT5G13080 | 1.1 | 3 | 166, 1474, 2210 | WRKY75 (WRKY DNA-BINDING PROTEIN 75) |
| 258975_at | AT3G01970 | 1.1 | 2 | 327, 2886 | WRKY45 (WRKY DNA-binding protein 45); transcription factor |
| 258751_at | AT3G05890 | 1.1 | 2 | 1783, 2908 | RCI2B (RARE-COLD-INDUCIBLE 2B) |
| 257644_at | AT3G25780 | 1.1 | 2 | 500, 2946 | AOC3 (ALLENE OXIDE CYCLASE 3) |
| 252114_at | AT3G51450 | 1.1 | 2 | 1863, 2971 | strictosidine synthase family protein |
| 254592_at | AT4G18880 | 1.1 | 2 | 75, 497 | AT-HSFA4A |
| 253257_at | AT4G34390 | 1.1 | 2 | 250, 2039 | XLG2 (EXTRA-LARGE GTP-BINDING PROTEIN 2); signal transducer |
| 261692_at | AT1G08450 | 1.1 | 1 | 2451 | CRT3 (CALRETICULIN 3); calcium ion binding |
| 259518_at | AT1G20510 | 1.1 | 1 | 2861 | OPCL1 (OPC-8:0 COA LIGASE1); 4-coumarate-CoA ligase |
| 262357_at | AT1G73040 | 1.1 | 1 | 13 | jacalin lectin family protein |
| 266071_at | AT2G18680 | 1.1 | 1 | 2080 | similar to unknown protein |
| 267548_at | AT2G32660 | 1.1 | 1 | 2322 | disease resistance family protein / LRR family protein |
| 258809_at | AT3G04070 | 1.1 | 1 | 232 | ANAC047 (Arabidopsis NAC domain containing protein 47) |
| 256883_at | AT3G26440 | 1.1 | 1 | 2882 | similar to unknown protein |
| 258259_s_at | AT3G26820 | 1.1 | 1 | 1185 | esterase/lipase/thioesterase family protein |
| 258259_s_at | AT3G26840 | 1.1 | 1 | 2454 | esterase/lipase/thioesterase family protein |
| 254833_s_at | AT4G12280 | 1.1 | 1 | 91 | copper amine oxidase family protein |
| 254660_at | AT4G18250 | 1.1 | 1 | 1116 | receptor serine/threonine kinase, putative |
| 254408_at | AT4G21390 | 1.1 | 1 | 2121 | B120; protein kinase/ sugar binding |
| 253124_at | AT4G36030 | 1.1 | 1 | 1354 | armadillo/beta-catenin repeat family protein |
| 252862_at | AT4G39830 | 1.1 | 1 | 811 | L-ascorbate oxidase, putative |
| 250287_at | AT5G13330 | 1.1 | 1 | 1594 | RAP2.6L (related to AP2 6L); DNA binding |
| 246495_at | AT5G16200 | 1.1 | 1 | 2798 | 50S ribosomal protein-related |
| 246831_at | AT5G26340 | 1.1 | 1 | 821 | MSS1 (SUGAR TRANSPORT PROTEIN 13); |
| 248889_at | AT5G46230 | 1.1 | 1 | 562 | similar to unknown protein |
| 248794_at | AT5G47220 | 1.1 | 1 | 2829 | ATERF-2/ATERF2/ERF2 (ETHYLENE RESPONSE FACTOR 2) |
| 247240_at | AT5G64660 | 1.1 | 1 | 816 | U-box domain-containing protein |
| 257591_at | AT3G24900 | 1.0 | 3 | 681, 1197, 1226 | disease resistance family protein / LRR family protein |
| 252133_at | AT3G50900 | 1.0 | 3 | 1556, 1621, 1811 | similar to unknown protein |
| 265028_at | AT1G24530 | 1.0 | 2 | 2010, 2921 | transducin family protein / WD-40 repeat family protein |
| 255751_at | AT1G31950 | 1.0 | 2 | 535, 2742 | terpene synthase/cyclase family protein |
| 245731_at | AT1G73500 | 1.0 | 2 | 873, 2923 | ATMKK9 (Arabidopsis thaliana MAP kinase kinase 9); kinase |
| 245777_at | AT1G73540 | 1.0 | 2 | 2488, 2783 | ATNUDT21 |
| 262211_at | AT1G74930 | 1.0 | 2 | 2825, 2899 | ORA47; DNA binding / transcription factor |
| 259979_at | AT1G76600 | 1.0 | 2 | 696, 2006 | similar to unknown protein |
| 265728_at | AT2G31990 | 1.0 | 2 | 809, 1448 | exostosin family protein |
| 258395_at | AT3G15500 | 1.0 | 2 | 2502, 2888 | ATNAC3 |
| 252474_at | AT3G46620 | 1.0 | 2 | 214, 2903 | zinc finger (C3HC4-type RING finger) family protein |
| 252470_at | AT3G46930 | 1.0 | 2 | 2441, 2462 | protein kinase family protein |
| 252234_at | AT3G49780 | 1.0 | 2 | 1820, 2326 | ATPSK4 (PHYTOSULFOKINE 4 PRECURSOR); growth factor |
| 251925_at | AT3G54000 | 1.0 | 2 | 2724, 2757 | similar to unknown protein |
| 261585_at | AT1G01010 | 1.0 | 1 | 909 | ANAC001 |
| 262608_at | AT1G14120 | 1.0 | 1 | 2895 | 2-oxoglutarate-dependent dioxygenase, putative |
| 256159_at | AT1G30135 | 1.0 | 1 | 2851 | JAZ8/TIFY5A (JASMONATE-ZIM-DOMAIN PROTEIN 8) |
| 260116_at | AT1G33960 | 1.0 | 1 | 570 | AIG1 (AVRRPT2-INDUCED GENE 1); GTP binding |
| 260943_at | AT1G45145 | 1.0 | 1 | 914 | ATTRX5 (thioredoxin H-type 5); thiol-disulfide exchange |
| 260410_at | AT1G69870 | 1.0 | 1 | 2900 | proton-dependent oligopeptide transport (POT) family protein |
| 260206_at | AT1G70740 | 1.0 | 1 | 351 | protein kinase family protein |
| 267178_at | AT2G37750 | 1.0 | 1 | 2945 | unknown protein |
| 267168_at | AT2G37770 | 1.0 | 1 | 1179 | aldo/keto reductase family protein |
| 257536_at | AT3G02800 | 1.0 | 1 | 2919 | phosphoprotein phosphatase |
| 258757_at | AT3G10910 | 1.0 | 1 | 1213 | zinc finger (C3HC4-type RING finger) family protein] |
| 258757_at | AT3G10912 | 1.0 | 1 | 1213 | CPuORF63 |
| 258362_at | AT3G14280 | 1.0 | 1 | 2298 | similar to hypothetical protein |
| 256756_at | AT3G25610 | 1.0 | 1 | 673 | haloacid dehalogenase-like hydrolase family protein |
| 252592_at | AT3G45640 | 1.0 | 1 | 1876 | ATMPK3 (MITOGEN-ACTIVATED PROTEIN KINASE 3) |
| 252403_at | AT3G48080 | 1.0 | 1 | 249 | lipase class 3 family protein / disease resistance protein |
| 252009_at | AT3G52800 | 1.0 | 1 | 1175 | zinc finger (AN1-like) family protein |
| 251722_at | AT3G56200 | 1.0 | 1 | 2447 | amino acid transporter family protein |
| 251293_at | AT3G61930 | 1.0 | 1 | 1190 | unknown protein |
| 255595_at | AT4G01700 | 1.0 | 1 | 1936 | chitinase, putative |
| 253193_at | AT4G35380 | 1.0 | 1 | 70 | guanine nucleotide exchange family protein |
| 253046_at | AT4G37370 | 1.0 | 1 | 2769 | CYP81D8 |
| 245711_at | AT5G04340 | 1.0 | 1 | 2766 | C2H2 (ZINC FINGER OF ARABIDOPSIS THALIANA 6) |
| 250024_at | AT5G18270 | 1.0 | 1 | 1877 | ANAC087; transcription factor |
| 246777_at | AT5G27420 | 1.0 | 1 | 2875 | zinc finger (C3HC4-type RING finger) family protein |
| 249197_at | AT5G42380 | 1.0 | 1 | 2655 | CML37/CML39; calcium ion binding |
| 247678_at | AT5G59520 | 1.0 | 1 | 298 | ZIP2 (ZINC TRANSPORTER 2 PRECURSOR); |

^a^ GeneChip ATH1 (Afymetrix) was employed with its standard protocol. Gene were sorted high to low in the log ratio of up-regulation and in the number of G-box sequences of each gene.

^b^ Probe ID of GeneChip ATH1.

^c^ Log_2_ ratio, *e.g.*, 1.0 indicating a 2-fold increase in the transcript level. Averages from three experimental replicates were employed for the calculation.

^d^ Number of nucleotides in direction to upstream from transcript initiation site.
