# Supplementary material for: bHLH106 Integrates Functions of Multiple Genes through Their G-Box to Confer Salt Tolerance on Arabidopsis
Source: PLoS One. 2015 May 15;10(5):e0126872. doi: 10.1371/journal.pone.0126872 (PMC4433118; doi:10.1371/journal.pone.0126872)
Supplement: S2 Table — (DOCX) [file pone.0126872.s007.docx]

**Table S2.** Genes satisfying both criteria of presence of G-box in promoters and down-regulation in OX Lines

| Probe ^b^ | Atg Number | Log ^c^  Ratio | G-Box | Position ^d^ | Annotation |
| --- | --- | --- | --- | --- | --- |
| 263549_at | AT2G21650 | -2.6 | 1 | 2566 | MEE3 (maternal effect embryo arrest 3); |
| 252965_at | AT4G38860 | -2.3 | 1 | 584 | auxin-responsive protein, putative |
| 256096_at | AT1G13650 | -1.9 | 1 | 2934 | similar to 18S pre-ribosomal assembly protein gar2-related |
| 245624_at | AT4G14090 | -1.7 | 1 | 1868 | UDP-glucoronosyl/UDP-glucosyl transferase family protein |
| 262236_at | AT1G48330 | -1.6 | 1 | 2775 | similar to unknown protein |
| 264343_at | AT1G11850 | -1.5 | 1 | 2537 | unknown protein |
| 259391_s_at | AT1G06350 | -1.4 | 2 | 274, 1080 | fatty acid desaturase family protein |
| 251363_at | AT3G61250 | -1.4 | 1 | 1759 | AtMYB17 (myb domain protein 17); |
| 252972_at | AT4G38840 | -1.4 | 1 | 704 | auxin-responsive protein, putative |
| 248185_at | AT5G54060 | -1.3 | 3 | 2281, 2803, 2846 | UF3GT (UDP-GLUCOSE:FLAVONOID 3-OGLUCOSYLTRANSFERASE); |
| 266899_at | AT2G34620 | -1.3 | 2 | 2849, 2937 | mitochondrial transcription termination factor-related /mTERF-related |
| 255450_at | AT4G02850 | -1.3 | 1 | 1351 | phenazine biosynthesis PhzC/PhzF family protein |
| 253416_at | AT4G33070 | -1.3 | 1 | 1328 | pyruvate decarboxylase, putative |
| 252916_at | AT4G38950 | -1.3 | 1 | 2816 | kinesin motor family protein |
| 246540_at | AT5G15600 | -1.1 | 2 | 2899, 2949 | SP1L4 (SPIRAL1-LIKE4) |
| 260210_at | AT1G74420 | -1.1 | 1 | 1497 | FUT3 (fucosyltransferase 3); |
| 252381_s_at | AT3G47750 | -1.1 | 1 | 2883 | ATPase, coupled to transmembrane movement of substances |
| 251586_at | AT3G58070 | -1.1 | 1 | 864 | GIS (GLABROUS INFLORESCENCE STEMS) |
| 245353_at | AT4G16000 | -1.1 | 1 | 2547 | similar to unknown protein |
| 250598_at | AT5G07690 | -1.1 | 1 | 2108 | MYB29 (myb domain protein 29); DNA binding /transcription factor |
| 249215_at | AT5G42800 | -1.0 | 4 | 2167, 2187, 2192, 2873 | DFR (DIHYDROFLAVONOL 4-REDUCTASE) |
| 263168_at | AT1G03020 | -1.0 | 2 | 52, 1922 | glutaredoxin family protein |
| 264990_at | AT1G27210 | -1.0 | 2 | 1687, 1732 | ARM repeat superfamily protein |
| 267158_at | AT2G37640 | -1.0 | 2 | 500, 1074 | ATEXPA3 (ARABIDOPSIS THALIANA EXPANSIN A3) |
| 251302_at | AT3G61970 | -1.0 | 2 | 1263, 1495 | NGA2 (NGATHA2); transcription factor |
| 255265_at | AT4G05190 | -1.0 | 2 | 1215, 1283 | ATK5 (Arabidopsis thaliana kinesin 5); microtubule motor |
| 245479_at | AT4G16140 | -1.0 | 2 | 2399, 2484 | proline-rich family protein |
| 250099_at | AT5G17300 | -1.0 | 2 | 543, 2800 | myb family transcription factor |
| 260297_at | AT1G80280 | -1.0 | 1 | 1988 | hydrolase, alpha/beta fold family protein |
| 258497_at | AT3G02380 | -1 | 1 | 2924 | COL2 (CONSTANS-LIKE 2); transcription factor/ zinc ion binding |
| 257191_at | AT3G13175 | -1.0 | 1 | 277 | similar to unnamed protein product |
| 258196_at | AT3G13980 | -1.0 | 1 | 2889 | similar to unknown protein |
| 252412_at | AT3G47295 | -1.0 | 1 | 2988 | unknown protein |
| 253836_at | AT4G27840 | -1.0 | 1 | 2847 | similar to vesicle-associated membrane protein-related |
| 248759_at | AT5G47610 | -1.0 | 1 | 2022 | zinc finger (C3HC4-type RING finger) family protein |
| 248311_at | AT5G52570 | -1.0 | 1 | 2726 | BETA-OHASE 2 (BETA-CAROTENE HYDROXYLASE 2); |

^a^ GeneChip ATH1 (Afymetrix) was employed with its standard protocol. Gene were sorted low to high in the log ratio of down-regulation and high to low in the number of G-box sequences of each gene.

^b^ Probe ID of GeneChip ATH1.

^c^ Log_2_ ratio, *e.g.*, -1.0 indicating a decrease in the transcript level of one half. Averages from three experimental replicates were employed for the calculation.

^d^ Number of nucleotides in direction to upstream from transcript initiation site.
