# Supplementary material for: bHLH106 Integrates Functions of Multiple Genes through Their G-Box to Confer Salt Tolerance on Arabidopsis
Source: PLoS One. 2015 May 15;10(5):e0126872. doi: 10.1371/journal.pone.0126872 (PMC4433118; doi:10.1371/journal.pone.0126872)
Supplement: S3 Table — (DOCX) [file pone.0126872.s008.docx]

**Table S3.**  Functional classification of genes satisfying both criteria of presence of G-box in promoters and up-regulation in OX Lines ^a^

Salt-Stress Regulated Genes

| Atg Number | Log ^b^  ratio | G-box | Position ^c^ | TAIR description |
| --- | --- | --- | --- | --- |
| AT1G61340 | 2.4 | 1 | 2759 | ATFBS1: Encodes a F-box protein induced by various biotic or abiotic stress. |
| AT3G08720 | 1.8 | 2 | 1779, 2916 | ARABIDOPSIS THALIANA PROTEIN KINASE 19 |
| AT3G51860 | 1.7 | 3 | 1057, 1654, 2302 | Cation exchanger 3 (CAX3) |
| AT1G27730 | 1.6 | 1 | 2760 | SALT TOLERANCE ZINC FINGER, STZ, ZAT10 |
| AT5G66640 | 1.6 | 1 | 2035 | DA1-related protein 3 (DAR3) |
| AT5G66640 | 1.6 | 1 | 2035 | DA1-RELATED PROTEIN 3, DAR3 |
| AT5G67450 | 1.5 | 1 | 1995 | ZINC-FINGER PROTEIN 1 |
| AT1G78410 | 1.3 | 2 | 2149, 2198 | VQ motif-containing protein |
| AT4G12720 | 1.3 | 1 | 164 | ARABIDOPSIS THALIANA NUDIX HYDROLASE HOMOLOG 7 |
| AT3G55980 | 1.2 | 1 | 2496 | Salt-inducible zinc finger 1 (SZF1) |
| AT4G18880 | 1.1 | 2 | 75, 497 | Arabidopsis thaliana heat shock transcription factor A4A |
| AT4G39830 | 1.1 | 1 | 811 | Cupredoxin superfamily protein |
| AT5G26340 | 1.1 | 1 | 821 | SUGAR TRANSPORT PROTEIN 13 |
| AT1G74930 | 1.0 | 2 | 2825, 2899 | Encodes a member of the DREB subfamily A-5 of ERF/AP2 transcription factor family |
| AT1G69870 | 1.0 | 1 | 2900 | Encodes a low affinity nitrate transporter NRT1.7 |
| AT3G25610 | 1.0 | 1 | 673 | ATPase E1-E2 type family protein |

Cold/Drought-Responsive Genes

| Atg Number | Log ^b^  ratio | G-box | Position ^c^ | TAIR description |
| --- | --- | --- | --- | --- |
| AT2G18660 | 3.0 | 1 | 793 | Encodes PNP-A (Plant Natriuretic Peptide A) |
| AT2G35930 | 2.6 | 2 | 2339, 2727 | ATPUB23: Encodes a cytoplasmically localized U-box domain containing E3 ubiquitin ligase |
| AT1G12610 | 1.7 | 2 | 1750, 1792 | Encodes a member of the DREB subfamily A-1 of ERF/AP2 transcription factor family (DDF1) |
| AT1G01560 | 1.6 | 1 | 1413 | Member of MAP Kinase |
| AT5G67450 | 1.5 | 1 | 1995 | Encodes zinc-finger protein |
| AT4G25470 | 1.3 | 1 | 2884 | ATCBF2, C-REPEAT/DRE BINDING FACTOR 2, CBF2 |
| AT4G02330 | 1.2 | 1 | 23 | ATPME41: Encodes a pectin methylesterase that is sensitive to chilling stress and brassinosteroid regulation. |
| AT3G05890 | 1.1 | 2 | 1783, 2908 | RARE-COLD-INDUCIBLE 2B, RCI2B |
| AT5G13330 | 1.1 | 1 | 1594 | Encodes a member of the ERF (ethylene response factor) subfamily B-4 of ERF/AP2 transcription factor family |
| AT5G64660 | 1.1 | 1 | 816 | "CYS, MET, PRO, AND GLY PROTEIN 2", ATCMPG2, CMPG2 |
| AT1G24530 | 1.0 | 2 | 2010, 2921 | Transducin/WD40 repeat-like superfamily protein |
| AT2G37770 | 1.0 | 1 | 1179 | AKR4C9, ALDO-KETO REDUCTASE FAMILY 4 MEMBER C9 |
| AT5G04340 | 1.0 | 1 | 2766 | C2H2, COLD INDUCED ZINC FINGER PROTEIN 2, |

Iron Ion Transport / Response / Regulated Genes

| Atg Number | Log ^b^  ratio | G-box | Position ^c^ | TAIR description |
| --- | --- | --- | --- | --- |
| AT4G26010 | 2.8 | 1 | 1079 | Peroxidase superfamily protein |
| AT4G19690 | 1.9 | 1 | 2301 | ARABIDOPSIS IRON-REGULATED TRANSPORTER 1 |
| AT3G01190 | 1.8 | 1 | 1028 | Peroxidase superfamily protein |
| AT5G59090 | 1.7 | 1 | 267 | ATSBT4.12, SBT4.12, SUBTILASE |
| AT1G22440 | 1.5 | 2 | 745, 2672 | Zinc-binding alcohol dehydrogenase family protein |
| AT2G21045 | 1.4 | 1 | 1537 | Rhodanese/Cell cycle control phosphatase superfamily protein |
| AT3G25930 | 1.2 | 1 | 222 | Adenine nucleotide alpha hydrolases-like superfamily protein |
| AT5G59520 | 1.0 | 1 | 298 | ATZIP2, ZIP2, ZRT/IRT-LIKE PROTEIN 2 |

Protein Kinases

| Atg Number | Log ^b^  ratio | G-box | Position ^c^ | TAIR description |
| --- | --- | --- | --- | --- |
| AT3G08720 | 1.8 | 2 | 1779, 2916 | ARABIDOPSIS THALIANA PROTEIN KINASE 19 |
| AT1G01560 | 1.6 | 1 | 1413 | ATMPK11, MAP KINASE 11, MPK11 |
| AT5G66210 | 1.4 | 1 | 2352 | CALCIUM-DEPENDENT PROTEIN KINASE 28, CPK28 |
| AT1G14370 | 1.2 | 2 | 620, 1404 | Encodes protein kinase APK2a. Protein is N-myristoylated |
| AT3G51450 | 1.1 | 2 | 1863, 2971 | Calcium-dependent phosphotriesterase superfamily protein |
| AT4G18250 | 1.1 | 1 | 1116 | Receptor serine/threonine kinase |
| AT1G73500 | 1.0 | 2 | 873, 2923 | ATMKK9, MAP KINASE KINASE 9, MKK9 |
| AT3G46930 | 1.0 | 2 | 2441, 2462 | Protein kinase superfamily protein |
| AT1G70740 | 1.0 | 1 | 351 | Protein kinase superfamily protein |
| AT3G45640 | 1.0 | 1 | 1876 | ATMAPK3, ATMPK3, MITOGEN-ACTIVATED PROTEIN KINASE 3, |

ANAC Transcription Factors

| Atg Number | Log ^b^  ratio | G-box | Position ^c^ | TAIR description |
| --- | --- | --- | --- | --- |
| AT5G39610 | 1.6 | 1 | 1764 | ANAC092, ARABIDOPSIS NAC DOMAIN CONTAINING PROTEIN 92 |
| AT3G04070 | 1.1 | 1 | 232 | ANAC047, NAC DOMAIN CONTAINING PROTEIN 47, NAC047 |
| AT3G15500 | 1.0 | 2 | 2502, 2888 | ANAC055, ATNAC3, NAC DOMAIN CONTAINING PROTEIN 3 |
| AT1G01010 | 1.0 | 1 | 909 | ANAC001, NAC DOMAIN CONTAINING PROTEIN 1, NAC001 |
| AT5G18270 | 1.0 | 1 | 1877 | ANAC087, ARABIDOPSIS NAC DOMAIN CONTAINING PROTEIN 87 |

Zn-Finger Proteins

| Atg Number | Log ^b^  ratio | G-box | Position ^c^ | TAIR description |
| --- | --- | --- | --- | --- |
| AT5G59550 | 4.2 | 1 | 2979 | Encodes an ABA- and drought-induced RING-DUF1117 gene |
| AT4G14365 | 1.8 | 1 | 85 | XB3 ORTHOLOG 4 IN ARABIDOPSIS THALIANA, XBAT34 |
| AT1G66500 | 1.6 | 1 | 2915 | Pre-mRNA cleavage complex II; |
| AT3G28210 | 1.6 | 1 | 947 | PMZ, SAP12, STRESS-ASSOCIATED PROTEIN 12 |
| AT5G67450 | 1.5 | 1 | 1995 | AZF1, ZF1, ZINC-FINGER PROTEIN 1 |
| AT3G10910 | 1.0 | 1 | 1213 | DAF-LIKE GENE 1, DAFL1 |
| AT3G52800 | 1.0 | 1 | 1175 | A20/AN1-like zinc finger family protein |
| AT5G27420 | 1.0 | 1 | 2875 | ARABIDOPSIS TOXICOS EN LEVADURA 31, ATL31 |

ABA / Salicylic Acid-Responsive Genes

| Atg Number | Log ^b^  ratio | G-box | Position ^c^ | TAIR description |
| --- | --- | --- | --- | --- |
| AT3G50930 | 2.9 | 1 | 209 | BCS1, CYTOCHROME BC1 SYNTHESIS |
| AT1G05575 | 1.8 | 1 | 1807 | Unknown protein |
| AT1G73540 | 1.0 | 2 | 2488, 2783 | ATNUDT21, NUDIX HYDROLASE HOMOLOG 21, NUDT21 |
| AT3G46620 | 1.0 | 2 | 214, 2903 | Encodes an ABA- and drought-induced RING-DUF1117 gene |
| AT3G02800 | 1.0 | 1 | 2919 | Encodes an atypical dual-speciﬁcity phosphatase |

Ethylene-Responsive Genes

| Atg Number | Log ^b^  ratio | G-box | Position ^c^ | TAIR description |
| --- | --- | --- | --- | --- |
| AT4G34410 | 2.5 | 4 | 993, 1830, 2581, 2867 | REDOX RESPONSIVE TRANSCRIPTION FACTOR 1, RRTF1 |
| AT1G28370 | 2.1 | 1 | 2658 | ATERF11, ERF DOMAIN PROTEIN 11, ERF11 |
| AT2G44840 | 2.1 | 1 | 1985 | ETHYLENE-RESPONSIVE ELEMENT BINDING FACTOR 13 |
| AT1G20350 | 1.8 | 2 | 28, 806 | ATTIM17-1, TIM17-1, TRANSLOCASE INNER MEMBRANE SUBUNIT 17-1 |
| AT1G09080 | 1.8 | 1 | 2816 | BINDING PROTEIN 3, BIP3 |
| AT4G17490 | 1.5 | 1 | 105 | ETHYLENE RESPONSIVE ELEMENT BINDING FACTOR 6 |
| AT4G17500 | 1.3 | 4 | 434, 955, 2344, 2793 | ETHYLENE RESPONSIVE ELEMENT BINDING FACTOR 1 |
| AT5G25250 | 1.2 | 1 | 526 | Encodes a protein that is involved in a membrane microdomain-dependent |
| AT5G13080 | 1.1 | 3 | 166, 1474, 2210 | ARABIDOPSIS THALIANA WRKY DNA-BINDING PROTEIN 75 |
| AT5G47220 | 1.1 | 1 | 2829 | ETHYLENE RESPONSIVE ELEMENT BINDING FACTOR 2 |

Cytochrome P450 Genes

| Atg Number | Log ^b^  ratio | G-box | Position ^c^ | TAIR description |
| --- | --- | --- | --- | --- |
| AT2G27690 | 2.4 | 1 | 947 | "CYTOCHROME P450, FAMILY 94, SUBFAMILY C, POLYPEPTIDE 1", CYP94C1 |
| AT4G13290 | 1.8 | 1 | 2446 | CYTOCHROME P450, FAMILY 71, SUBFAMILY A, POLYPEPTIDE 19", CYP71A19 |
| AT5G45340 | 1.7 | 2 | 2098, 2858 | CYTOCHROME P450, FAMILY 707, SUBFAMILY A, POLYPEPTIDE 3", CYP707A3 |
| AT3G48520 | 1.5 | 1 | 1769 | CYP94B3, CYTOCHROME P450, FAMILY 94, SUBFAMILY B, POLYPEPTIDE 3 |
| AT3G26210 | 1.4 | 1 | 2307 | CYTOCHROME P450, FAMILY 71, SUBFAMILY B, POLYPEPTIDE 23", CYP71B23 |
| AT4G13310 | 1.4 | 1 | 2356 | CYTOCHROME P450, FAMILY 71, SUBFAMILY A, POLYPEPTIDE 20", CYP71A20 |
| AT4G37370 | 1.0 | 1 | 2769 | CYTOCHROME P450, FAMILY 81, SUBFAMILY D, POLYPEPTIDE 8", CYP81D8 |

Protein Phosphatase 2C Genes

| Atg Number | Log ^b^  ratio | G-box | Position ^c^ | TAIR description |
| --- | --- | --- | --- | --- |
| AT2G05050 | 1.3 | 1 | 2853 | Protein phosphatase 2C family protein |
| AT3G27140 | 1.3 | 1 | 2853 | Protein phosphatase 2C family protein |
| AT4G08260 | 1.3 | 1 | 2853 | Protein phosphatase 2C family protein |

Jasmonic Acid Biosynthesis / Metabolism / Responsive Genes

| Atg Number | Log ^b^  ratio | G-box | Position ^c^ | TAIR description |
| --- | --- | --- | --- | --- |
| AT1G19180 | 2.0 | 3 | 149, 1840, 2936 | ATJAZ1, JASMONATE-ZIM-DOMAIN PROTEIN 1, JAZ1, TIFY10A |
| AT1G17380 | 1.7 | 1 | 2113 | JASMONATE-ZIM-DOMAIN PROTEIN 5, JAZ5, TIFY11A |
| AT3G55970 | 1.3 | 1 | 1170 | ATJRG21, JASMONATE-REGULATED GENE 21, JRG21 |
| AT1G19640 | 1.2 | 2 | 730, 794 | ASMONIC ACID CARBOXYL METHYLTRANSFERASE, JMT |
| AT3G56710 | 1.1 | 5 | 567, 1193, 1539, 1863, 2677 | SIB1, SIGMA FACTOR BINDING PROTEIN 1 |
| AT3G25780 | 1.1 | 2 | 500, 2946 | ALLENE OXIDE CYCLASE 3, AOC3 |
| AT1G20510 | 1.1 | 1 | 2861 | OPC-8:0 COA LIGASE1, OPCL1 |
| AT1G30135 | 1.0 | 1 | 2851 | JASMONATE-ZIM-DOMAIN PROTEIN 8, JAZ8, TIFY5A |

WRKY family Transcription Factors

| Atg Number | Log ^b^  ratio | G-box | Position ^c^ | TAIR description |
| --- | --- | --- | --- | --- |
| AT5G22570 | 1.2 | 1 | 1166 | RABIDOPSIS THALIANA WRKY DNA-BINDING PROTEIN 38 |
| AT5G13080 | 1.1 | 3 | 166, 1474, 2210 | ARABIDOPSIS THALIANA WRKY DNA-BINDING PROTEIN 75 |
| AT3G01970 | 1.1 | 2 | 327, 2886 | ATWRKY45, WRKY DNA-BINDING PROTEIN 45, WRKY45 |

^a^ GeneChip ATH1 (Afymetrix) was employed with its standard protocol. Gene were sorted high to low in the log ratio of up-regulation and in the number of G-box sequences of each gene.

^b^ Log_2_ ratio, *e.g.*, 1.0 indicating a 2-fold increase in the transcript level. Averages from three experimental replicates were employed for the calculation.

^c^ Number of nucleotides in direction to upstream from transcript initiation site.
