# Supplementary material for: bHLH106 Integrates Functions of Multiple Genes through Their G-Box to Confer Salt Tolerance on Arabidopsis
Source: PLoS One. 2015 May 15;10(5):e0126872. doi: 10.1371/journal.pone.0126872 (PMC4433118; doi:10.1371/journal.pone.0126872)
Supplement: S4 Table — (DOCX) [file pone.0126872.s009.docx]

**Table S4.** Salt-, cold-, or drought-responsive genes which also satisfied both criteria of presence of G-box in promoters and down-regulation in OX Lines ^a^

| Atg Number | Log ^b^  ratio | G-box | Position ^c^ | TAIR description |
| --- | --- | --- | --- | --- |
| AT2G21650 | -2.6 | 1 | 2566 | ARABIDOPSIS RAD-LIKE 2 |
| AT4G38860 | -2.3 | 1 | 584 | SAUR-like auxin-responsive protein family |
| AT1G13650 | -1.9 | 1 | 2934 | 8S pre-ribosomal assembly protein gar2-related |
| AT4G14090 | -1.7 | 1 | 1868 | anthocyanidin 5-O-glucosyltransferase |
| AT4G38840 | -1.4 | 1 | 704 | SAUR-like auxin-responsive protein family |
| AT5G07690 | -1.1 | 1 | 2108 | MYB DOMAIN PROTEIN 29 |
| AT4G05190 | -1.0 | 2 | 1215, 1283 | ATK5 encodes a kinesin protein |
| AT5G17300 | -1.0 | 2 | 543, 2800 | Myb-like transcription factor |

^a^ GeneChip ATH1 (Afymetrix) was employed with its standard protocol. Gene were sorted low to high in the log ratio of down-regulation and high to low in the number of G-box sequences of each gene.

^b^ Log_2_ ratio, *e.g.*, -1.0 indicating a decrease in the transcript level of one half. Averages from three experimental replicates were employed for the calculation.

^c^ Number of nucleotides in direction to upstream from transcript initiation site.
