# Supplementary material for: bHLH106 Integrates Functions of Multiple Genes through Their G-Box to Confer Salt Tolerance on Arabidopsis
Source: PLoS One. 2015 May 15;10(5):e0126872. doi: 10.1371/journal.pone.0126872 (PMC4433118; doi:10.1371/journal.pone.0126872)
Supplement: S5 Table — (DOCX) [file pone.0126872.s010.docx]

**Table S5.** Probes for genes possessing G-box in their promoters ^a^

|  | Probe | Forward (5’-3’) ^b^ | Probe | Reverse (5’-3’) ^b^ |
| --- | --- | --- | --- | --- |
| 1 | At2g27730-F | GACCTAGTACACGTGTGTGTGTGT | At2g27730-R | ACACACACACACGTGTACTAGGTC |
| 2 | At1g18740-F | CATTTTCTCCACGTGGCACCCATT | At1g18740-R | AATGGGTGCCACGTGGAGAAAATG |
| 3 | At1g74450-F | TACATCTTCCACGTGCTGTCATGT | At1g74450-R | ACATGACAGCACGTGGAAGATGTA |
| 4 | At1g74450-F2 | ACGTCTCGCCACGTGGCCTCCGTT | At1g74450-R2 | AACGGAGGCCACGTGGCGAGACGT |
| 5 | At1g74450-F3 | TGTAATCCACACGTGCTCTCTACT | At1g74450-R3 | AGTAGAGAGCACGTGTGGATTACA |
| 6 | At3g27210-F | TTTTGGTGTCACGTGGCACTAATT | At3g27210-R | AATTAGTGCCACGTGACACCAAAA |
| 7 | At3g15210-F | AAAGAAAAACACGTGGCAAAAGAT | At3g15210-R | ATCTTTTGCCACGTGTTTTTCTTT |
| 8 | At1g66500-F | GCGCGTTGTCACGTGTTTTGGGCC | At1g66500-R | GGCCCAAAACACGTGACAACGCGC |
| 9 | At3g49530-F | ATGTAATGACACGTGGAAAGGAAT | At3g49530-R | ATTCCTTTCCACGTGTCATTACAT |
| 10 | At5g45340-F | GACGAGAGCACGTGTCCAAGAGCA | At5g45340-R | TGCTCTTGGACACGTGCTCTCGTC |
| 11 | At5g45340-F2 | GGGAAAACCCACGTGTGGTCATTG | At5g45340-R2 | CAATGACCACACGTGGGTTTTCCC |
| 12 | At5g27420-F | TGACCGTCCACGTGGCATTCAATA | At5g27420-R | TATTGAATGCCACGTGGACGGTCA |
| 13 | At1g32640-F | GTTTGGTCGCACGTGTATCACACG | At1g32640-R | CGTGTGATACACGTGCGACCAAAC |
| 14 | At1g63720-F | GCAGAAGCGCACGTGTGGGAGAAT | At1g63720-R | ATTCTCCCACACGTGCGCTTCTGC |
| 15 | At3g46620-F | CGGTGAGAACACGTGACAGTGTTT | At3g46620-R | AAACACTGTCACGTGTTCTCACCG |
| 16 | At5g59550-F | GTAAATAGACACGTGGTGAGTGTT | At5g59550-R | AACACTCACCACGTGTCTATTTAC |
| 17 | At1g52890-F1 | GAATTCTGACACGTGTGTCAGTTA | At1g52890-R1 | TAACTGACACACGTGTCAGAATTC |
| 18 | At1g52890-F2 | ATTTTATTTCACGTGTTTTTTGTT | At1g52890-R2 | AACAAAAAACACGTGAAATAAAAT |
| 19 | At1g52890-F3 | TGACGACACGTGTCCACGTGTCTATC | At1g52890-R3 | GATAGACACGTGGACACGTGTCGTCA |
| 20 | At5g05250-F | ATCAATAGGCACGTGATAATATGG | At5g05250-R | CCATATTATCACGTGCCTATTGAT |
| 21 | At5g05250-F2 | CGTGCTTACCACGTGTTTGCCACG | At5g05250-R2 | CGTGGCAAACACGTGGTAAGCACG |
| 22 | At5g05250-F3 | CTTATTGTCCACGTGGAATCATCT | At5g05250-R3 | AGATGATTCCACGTGGACAATAAG |
| 23 | At2g43010-F | AGAACTTGCCACGTGTCGTTCATT | At2g43010-R | AATGAACGACACGTGGCAAGTTCT |
| 24 | At1g74930-F  (DREBA-5) | ACAATCCGCCACGTGTCCCATCTT | At5g49730-F  (DREBA-5) | AAGATGGGACACGTGGCGGATTGT |
| 25 | At5g49730-F2  (DREBA-5) | GAATTAAATCACGTGGAAAACAAG | At5g49730-F2  (DREBA-5) | CTTGTTTTCCACGTGATTTAATTC |
| 26 | At5g49730-F  (DREBA-5) | TGTCATTAACACGTGAGAGTAACG | At5g49730-R  (DREBA-5) | CGTTACTCTCACGTGTTAATGACA |
| 27 | At1g73540-F | AGATAAGTTCACGTGAGAAATCCT | At1g73540-R | AGGATTTCTCACGTGAACTTATCT |
| 28 | At1g73540-F2 | CAACATGAACACGTGTTCATGCCA | At1g73540-R2 | TGGCATGAACACGTGTTCATGTTG |
| 29 | At2g40200-F | GCAAAAAAACACGTGTGTAAAAAT | At2g40200-R | ATTTTTACACACGTGTTTTTTTGC |
| 30 | At1g15580-F | ACAACCGAACACGTGTTACTCAAG | At1g15580-R | CTTGAGTAACACGTGTTCGGTTGT |
| 31 | At4g28720-F | TTTCTTCCCCACGTGGCTTCCTCT | At4g28720-R | AGAGGAAGCCACGTGGGGAAGAAA |
| 32 | At4g28720-F2 | TCATCCTCTCCACGTGGCTTCCATT | At4g28720-R2 | AATGGAAGCCACGTGGAGAGGATGA |
| 33 | At2g31980-F | TTAAGTCTCCACGTGAAGTGAGCC | At2g31980-R | GGCTCACTTCACGTGGAGACTTAA |
| 34 | At3g07350-F | ATCTCATGACACGTGGATGTCACA | At3g07350-R | TGTGACATCCACGTGTCATGAGAT |
| 35 | At3g28740-F | TCCATGTGACACGTGAACATTATG | At3g28740-R | CATAATGTTCACGTGTCACATGGA |
| 36 | At3g51450-F | CATTAAATACACGTGTTTAATTTA | At3g51450-R | TAAATTAAACACGTGTATTTAATG |
| 37 | At3g51450-F2 | CAAAAGTGACACGTGTACTTCTCA | At3g51450-R2 | TGAGAAGTACACGTGTCACTTTTG |
| 38 | At4g15210-F | GTTTTTAAGCACGTGGAGTAAATG | At4g15210-R | CATTTACTCCACGTGCTTAAAAAC |
| 39 | At3g50060-F | GTAGGTGTACACGTGTCGTGATCT | At3g50060-R | AGATCACGACACGTGTACACCTAC |
| 40 | At2g02810-F | ATTAGCTTCCACGTGGAACATCGA | At2g02810-R | TCGATGTTCCACGTGGAAGCTAAT |

|  | Probe | Forward (5’-3’) ^b^ | Probe | Reverse (5’-3’) ^b^ |
| --- | --- | --- | --- | --- |
| 41 | At2g40300-F | GATGAGAGACACGTGTACGTACGA | At2g40300-R | TCGTACGTACACGTGTCTCTCATC |
| 42 | At1g14360-F | TAGATTTTCCACGTGTAACTGTGT | At1g14360-R | ACACAGTTACACGTGGAAAATCTA |
| 43 | At1g14360-F2 | TTTTGTCCACACGTGATATGCAAT | At1g14360-R2 | ATTGCATATCACGTGTGGACAAAA |
| 44 | At3g20340-F | CCAACGTTCCACGTGTCCTTATTG | At3g20340-R | CAATAAGGACACGTGGAACGTTGG |
| 45 | At3g53670-F | AACACGAGACACGTGTATATTTCT | At3g53670-R | AGAAATATACACGTGTCTCGTGTT |
| 46 | At5g01600-F | ATTGAGATCCACGTGGAGGGTGGA | At5g01600-R | TCCACCCTCCACGTGGATCTCAAT |
| 47 | At5g37260-F | TGCTAATGACACGTGGAGGATTTC | At5g37260-R | GAAATCCTCCACGTGTCATTAGCA |
| 48 | At2g15490-F | TTAAGTGCACACGTGCGTGTATAT | At2g15490-R | ATATACACGCACGTGTGCACTTAA |
| 49 | At2g15480-F | CAACGTGCACACGTGTCTGTGTAT | At2g15480-R | ATACACAGACACGTGTGCACGTTG |
| 50 | At5g62480-F | AGATCAAGCCACGTGGCTAAATTA | At5g62480-R | TAATTTAGCCACGTGGCTTGATCT |
| 51 | At5g62480-F2 | AGTCACGGACACGTGTTCACAAGC | At5g62480-R2 | GCTTGTGAACACGTGTCCGTGACT |
| 52 | At2g29330-F | CTACCTAAACACGTGAACAGTAGT | At2g29330-R | ACTACTGTTCACGTGTTTAGGTAG |
| 53 | At4g25920-F | GTATTTGGTCACGTGAGCTGCGTC | At4g25920-R | GACGCAGCTCACGTGACCAAATAC |
| 54 | At1g23510-F | TGTATAGTCCACGTGAATTTTCCC | At1g23510-R | GGGAAAATTCACGTGGACTATACA |
| 55 | At2g17590-F | TGATAAGTACACGTGTCATTAATA | At2g17590-R | TATTAATGACACGTGTACTTATCA |
| 56 | At1g32320-F | CCCATTTGCCACGTGTCATGTCCT | At1g32320-R | AGGACATGACACGTGGCAAATGGG |
| 57 | At3g57260-F | CTCAAGACACACGTGTTTAATATG | At3g57260-R | CATATTAAACACGTGTGTCTTGAG |
| 58 | At1g48120-F | ATACGAAGACACGTGGCGTGAACC | At1g48120-R | GGTTCACGCCACGTGTCTTCGTAT |
| 59 | At1g13260-F | TCAATATCTCACGTGGACCCTTTA | At1g13260-R | TAAAGGGTCCACGTGAGATATTGA |
| 60 | At1g19250-F | ATTAGGGAGCACGTGCTTTTGAAT | At1g19250-R | ATTCAAAAGCACGTGCTCCCTAAT |

^a^ Genes were selected randomly among GeneChip data of *bHLH106*-KO and -OX lines.

^b^ G-box sequences are underlined.
